# TEpiNom: A computational framework integrating population data to prioritize *Plasmodium falciparum* T cell epitopes

**DOI:** 10.1016/j.vaccine.2026.128388

**Published:** 2026-03-02

**Authors:** Alexander J. Laurenson, Brian G. Pierce, Shannon Takala-Harrison, Matthew B. Laurens

**Affiliations:** a Center for Vaccine Development and Global Health, University of Maryland School of Medicine, Baltimore, MD, USA; b Molecular Microbiology and Immunology Program, Graduate Program in Life Sciences, University of Maryland School of Medicine, Baltimore, MD, USA; c University of Maryland Institute for Bioscience and Biotechnology Research, Rockville, MD, USA; d Department of Cell Biology and Molecular Genetics, University of Maryland, College Park, MD, USA

**Keywords:** Plasmodium falciparum, Malaria, Epitope prediction, Immunoinformatics, Multi-epitope vaccine

## Abstract

Developing a highly effective malaria vaccine remains challenging due to Plasmodium falciparum’s antigenic diversity and human leukocyte antigen (HLA) polymorphisms, which complicate antigen selection and limit immune protection. The first recommended malaria vaccine, RTS,S, provides partial, allele-specific protection with waning immunity, and recently developed R21 vaccine will likely encounter the same hurdles. To address these challenges, we developed a computational decision-support framework that integrates P. falciparum sequence diversity, predicted T cell epitope-HLA binding, and population-specific HLA allele frequencies from sub-Saharan Africa to prioritize conserved T cell epitopes for experimental evaluation. We analyzed 42 P. falciparum proteins, previously identified as vaccine candidates, generated consensus sequences from 18 African countries, and incorporated HLA allele frequencies from 24 sub-Saharan populations. CD8+ and CD4+ T cell epitopes were predicted using NetMHCpan-4.1 and NetMHCIIpan-4.1. Our tool, T cell Epitope Nomination (TEpiNom), applies integer linear programming to prioritize epitopes based on sequence conservation (>95%), predicted HLA binding affinity (median rank <10%), and breadth of HLA locus coverage, while minimizing redundancy across antigen targets. Using this framework, we identified 2265 MHC I and 1992 MHC II conserved epitopes spanning pre-erythrocytic, erythrocytic, and sexual stage proteins. Prioritized MHC I epitopes from pre-erythrocytic antigens FabZ, FabG, p36, and PKG achieved 100% predicted inter-locus MHC I coverage, and MHC II epitopes from pre-erythrocytic, erythrocytic, or sexual antigens provided 100% coverage for a given parasite life stage. In parallel, our search for epitope-dense regions identified short, conserved protein segments across all parasite life stages that independently provided complete predicted inter-locus coverage, highlighting compact targets with high HLA-promiscuous potential. Together, this study presents TEpiNom for the systematic prioritization of T cell epitopes and epitope-dense regions, to streamline preclinical malaria vaccine development by refining computational predictions into experimentally tractable candidates. The framework is adaptable for vaccine development against other diverse and evasive pathogens.

## Graphical Abstract

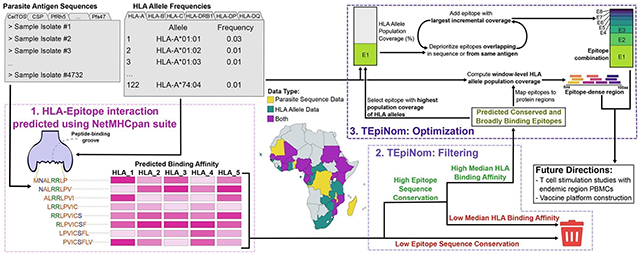

## 1. Introduction

Global malaria deaths declined from 897,000 in 2000 to 577,000 in 2015, but have since plateaued, with an estimated 597,000 malaria deaths in 2023, 76% of which occurred in children under five [1]. This stagnation occurred despite multiple public health interventions, underscoring the need for improved malaria control measures, including more effective vaccines.

The first licensed malaria vaccines, RTS,S/AS01 (RTS,S) and R21/Matrix-M (R21) target the Circumsporozoite Protein (CSP) expressed on circulating sporozoites during the pre-erythrocytic stage [2]. Though both demonstrate moderate efficacy, Phase III trials revealed at least three limitations: 1) RTS,S induces allele-specific immunity, 2) efficacy wanes over time without booster doses, and 3) protection is lower in younger children [2,3]. Long-term protection for the R21 vaccine remains unclear [4]. Despite public health benefit of these vaccines, these limitations highlight broader challenges for malaria vaccine development, as parasite genetic diversity limits coverage of monovalent vaccines and can drive allele-specific immune responses [5,6], while variation in antigen presentation across HLA haplotypes further constrains the breadth of vaccine-induced immunity [7]. Addressing these challenges is complicated by the enormous scale of the *Plasmodium falciparum* (*Pf*) proteome, which comprises approximately 5300 genes [8,9]. Advances in large-scale genome sequencing, characterization, and surveillance, including the MalariaGEN Pf7 database of over 16,000 from 33 countries, have enabled systematic characterization of parasite diversity, particularly in sub-Saharan Africa where most malaria deaths occur [1,8,10–12].

Subunit vaccines such as RTS,S and R21 rely on one haplotype sequence of a protein, making them vulnerable to vaccine escape when infecting strains harbor divergent sequences [5,6,13–15]. Analogous to the selective pressures that have driven antimalarial drug resistance, selective pressure may favor parasites that circumvent allele-specific vaccine-induced immunity [5]. Novel malaria vaccine approaches are needed to achieve broad and robust immune recognition.

Reverse vaccinology represents a viable approach by using genomic information to identify vaccine candidate antigens systematically [16]. Epitope-based vaccine development extends this approach by prioritizing epitopes based on sequence conservation, predicted HLA binding promiscuity (for T cell epitopes), and predicted surface accessibility (for B cell epitopes), for downstream investigation. Although still developing, this approach supports rational selection of epitopes and protein targets before experimental validation, streamlining preclinical workflows [17,18].

**Figure 1:**
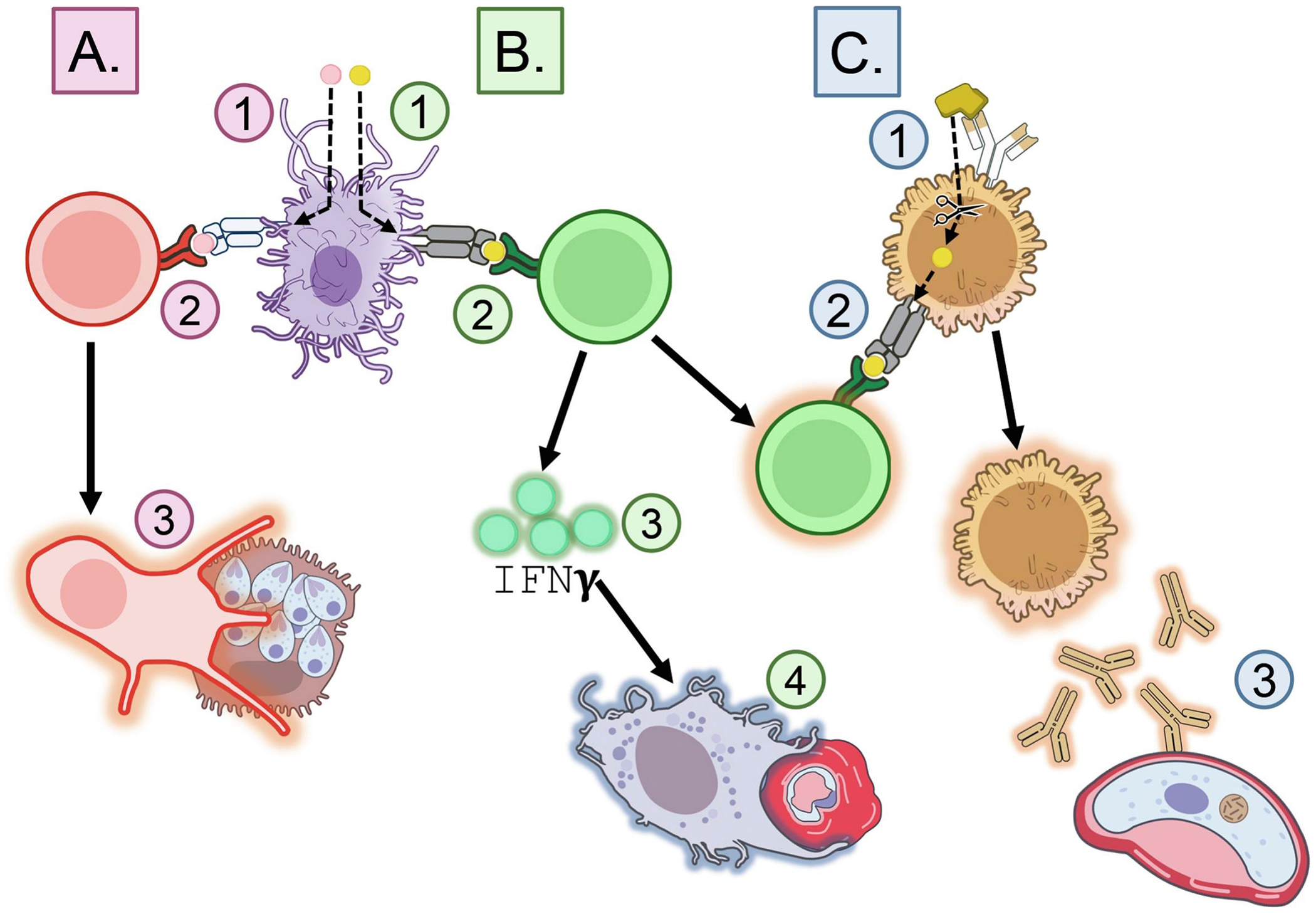
Epitope-induced immunity. **A. CD8+ Cytotoxic T cell activation.** 1. MHC I epitope uptake and processing by Antigen-Presenting Cell (APC). 2. Epitope cross-presentation on MHC I protein and binding to CD8+ T cell receptor. 3. Killing of sporozoite-infected hepatocytes by CD8+ Cytotoxic T cell. **B. CD4+ Helper T cell activation.** 1. MHC II epitope uptake and processing by APC. 2. Epitope presentation on MHC II protein and binding to CD4+ T cell receptor. 3. Production of IFNγ by CD4+ Helper T cell. 4. Killing of merozoite-infected red blood cells by IFNγ-activated macrophage. **C. B cell activation.** 1. B cell epitope binding to B cell receptor and antigen uptake. 2. Antigen processing and presentation of epitope on MHC II protein. 3. Activated CD4+ Helper T cell binds to epitope on MHC II and co-stimulates B cell. 4. Activated B cell produces antibodies specific to sexual stage protein. Art is licensed under Public Domain and available on NIH BIOART.

Cell-mediated immunity is critical to effective and durable protection, yet remains understudied compared to antibody responses [19]. During the pre-erythrocytic stage, CD8+ T cells kill infected hepatocytes (Fig. 1A) [20,21]. CD4+ T cells reinforce this response through the Th1-driven macrophage activation and B cell class switching (Fig. 1B) [22–27]. T follicular helper cells promote long-lived B cell responses (Fig. 1C) [28,29]. The described T cell roles motivate *Pf* T cell epitope investigation and inclusion in next-generation malaria vaccines.

Because peptide presentation by MHC I and II is a prerequisite for CD8+ and CD4+ T cell recognition, rational epitope selection includes identifying peptides with strong predicted binding to the MHC molecules encoded by the highly polymorphic human HLA loci [30,31]. Given that HLA allele frequencies differ across populations, incorporating population-specific HLA data is essential for achieving broad coverage. Computational models can reliably predict high affinity epitope HLA interactions at scale based on linear peptide features [32,33]. However, approaches relying on HLA supertypes may overlook substantial functional diversity, especially for alleles common in subSaharan Africa that fall outside canonical supertype groupings [34,35].

Recent work has demonstrated the feasibility of integrating *in silico* predictions with experimental validation to down-select *Pf* T cell epitopes [36]. Kotraiah et al. used prediction tools to identify HLA-DRB1 restricted CD4+ epitopes in five erythrocytic antigens and validated them with *in vitro* binding and *ex vivo* recall assays [36], illustrating the utility of immunoinformatic approaches for rational epitope selection. Despite the availability of parasite sequence data and HLA allele frequency datasets from endemic regions, larger-scale T epitope prediction remains underutilized. Although some tools, including TepiTool [37] and PopCover-2.0 [38], filter epitopes or optimize final epitope combinations, they lack features essential for malaria vaccine development under conditions of extensive parasite and HLA diversity. Specifically, they do not perform comprehensive global optimization of population coverage across all high-coverage epitope combinations while incorporating redundancy constraints, nor do they identify epitope-dense protein regions that maximize coverage within contiguous sequences.

**Figure 2:**
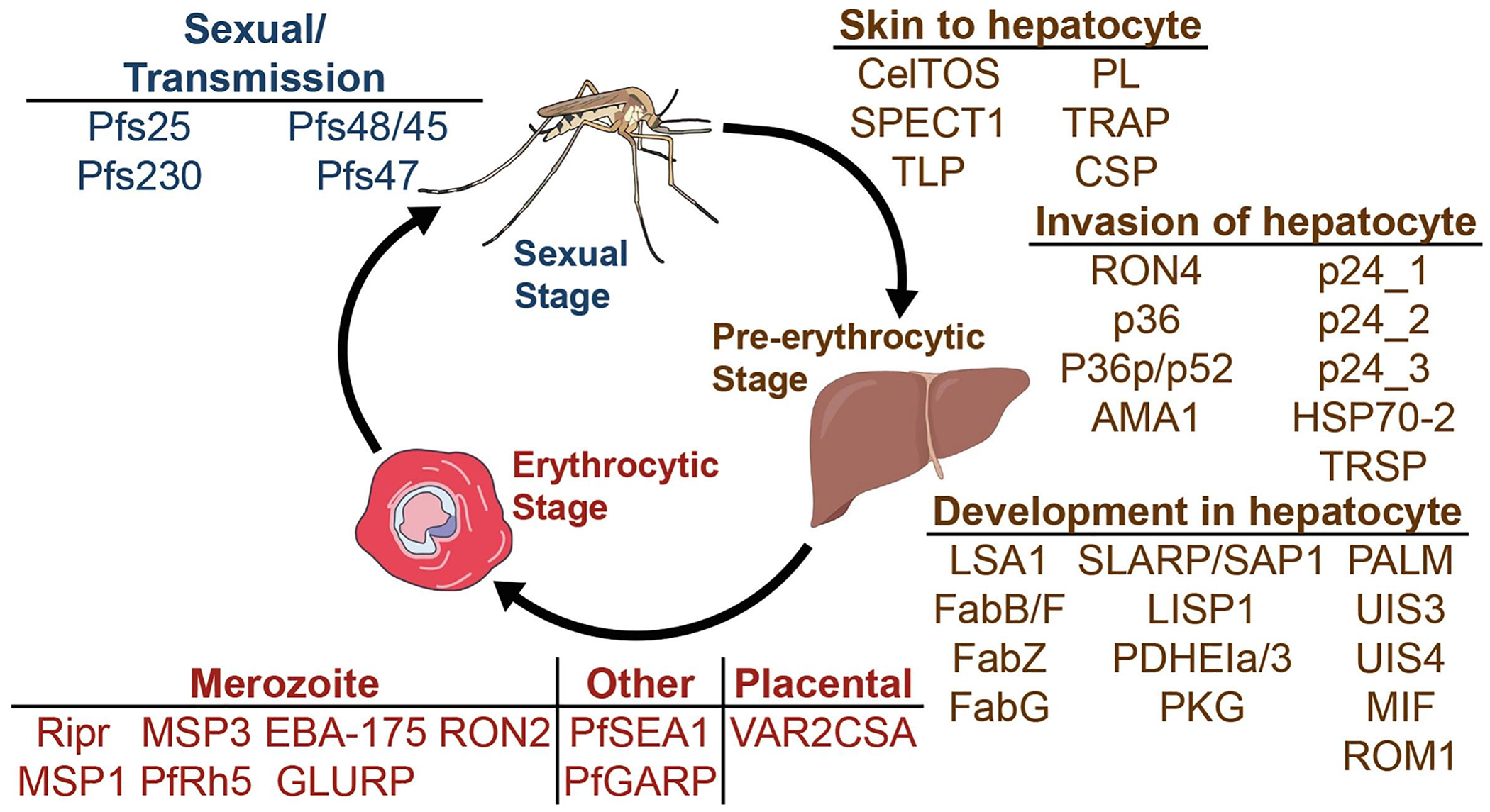
Vaccine candidates by malaria life stage. Liver, blood, and sexual/transmission stages of *Pf* and the vaccine candidates separated by stage and substage. Art is licensed under Public Domain and available on NIH BIOART and Bioicons.

This study describes an immunoinformatics-driven approach for prioritizing *Pf* T cell epitopes to support vaccine development. Using parasite protein sequences from field isolates and HLA allele frequency data from endemic African populations, we apply computational tools to predict and prioritize candidate T cell epitopes with strong predicted binding to diverse alleles (Fig. 2). We then introduce a down-selection framework integrating conservation, predicted binding affinity, and predicted HLA promiscuity to identify a manageable subset of high-priority candidates. Additionally, we incorporate a population coverage optimization step to select epitope combinations that maximize predicted population-level HLA presentation while minimizing protein redundancy in target epitope selection, and we identify epitope-dense regions with both favorable population coverage and high sequence conservation. This framework offers a decision-support system for refining large-scale epitope predictions into experimentally tractable candidates for downstream validation and vaccine design.

## 2. Methods

### 2.1. Selection of Pf antigens and retrieval of sequences

**Figure 3.**
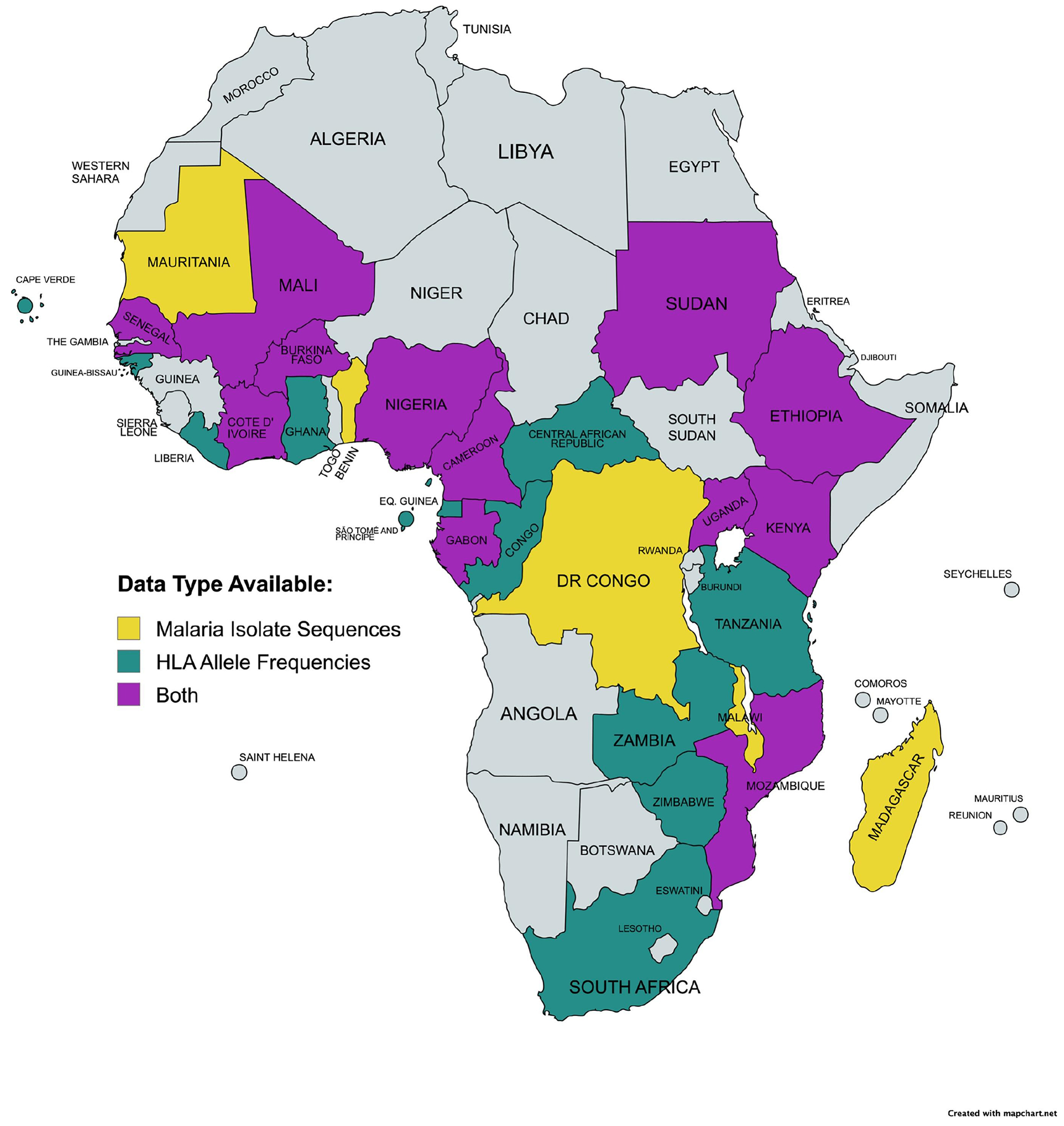
Parasite genomic and HLA data availability. Map of sub-Saharan African countries with MalariaGEN parasite data (yellow), HLA allele data (green), or both data types (purple). Created with MapChart.

Forty-two candidate proteins were selected based on prior studies demonstrating essential roles in *Pf* pathogenicity, particularly in invasion or survival (Supplementary Table 1; Fig. 2) [39–85]. Variant Call Files (VCFs) mapped to the Pf3D7 reference genome were acquired from MalariaGEN Pf7 and included variants for *Pf* isolates from sub-Saharan Africa (Fig. 3). Samples likely to represent monoclonal infections, through a within-host infection fixation index (F_WS_) ≥0.95, were selected for consensus sequence generation [12]. After normalization to the Pf3D7 reference genome, adjacent insertions and deletions within 5 base pairs were filtered, and sample-specific variants were incorporated into the Pf3D7 reference sequence backbone to generate full-length consensus sequences for each sample and then repeated for each antigen [87]. Sequences were translated using UCSC’s faTrans tool, and those with nonsense mutations were excluded [86]. The finalized datasets include protein sequences for each of the 42 vaccine candidates, comprising samples from 18 African countries, with a median of 3996 sample sequences per protein (Supplementary Tables 1–2, Fig. 3).

### 2.2. Selection of HLA alleles and population frequencies

HLA allele frequency data was obtained by searching the Allele Frequency Net Database (https://www.allelefrequencies.net/), a global repository for published MHC allele population frequency data, for HLA-A, HLA-B, HLA-C, HLA-DQ, HLA-DP, and HLA-DR alleles recorded within sub-Saharan African countries (Fig. 3) [87–89]. Extracted allele frequencies, representing the proportion of each allele within each population sample, were normalized within each HLA class to reflect the relative frequencies of HLA alleles. The finalized HLA allele dataset contains information from all 24 countries with published HLA datasets, encompassing frequencies for 748 unique HLA alleles (Fig. 3).

### 2.3. Diversity analysis of protein antigens

Sequences were clustered using CD-HIT (v4.8.1) at a 100% identity threshold to define unique haplotypes [90]. Haplotype diversity (*Hd*) was then calculated for each protein using the standard formula, $Hd=\frac{N}{N-1}\left(1-\sum_{i}x_{i}^{2}\right)$, where *N* is the number of sequences and *xi* is the frequency of each haplotype in the sample.

### 2.4. Prediction of T cell epitopes

T cell epitope binding to MHC I and II proteins was predicted using downloaded software for NetMHCpan-4.1 (https://services.healthtech.dtu.dk/services/NetMHCpan-4.1/) and NetMHCIIpan-4.1 (https://services.healthtech.dtu.dk/services/NetMHCIIpan-4.1/), neural network tools trained on both mass spectrometry binding affinity and eluted ligand data [33]. NetMHCpan-4.1 and NetMHCIIpan-4.1 were chosen specifically, as they offer high predictive accuracy across a broad HLA repertoire, with NetMHCpan-4.1 achieving a positive predictive value of about 88% and NetMHCIIpan-4.1 reaching about 80% for HLA-DR and -DP and about 60% for HLA-DQ, performing as well as or better than other models [33]. Their training on the most extensive HLA ligand and binding datasets to date enables reliable performance for both common and rare alleles, making these tools well suited for predicting T cell epitope binding across endemic region HLA profiles. Epitope predictions were performed against the HLA-A, HLA–B, HLA–C, HLA-DQ, HLA-DP, and HLA-DR alleles within the previously mentioned HLA allele dataset. CD8+ and CD4+ T cell epitopes were predicted using the default peptide lengths for MHC I and II epitopes, 8–11 and 15 amino acids, respectively. CD8+ T cell epitopes were predicted uniquely for pre-erythrocytic stage antigens and not for blood- or sexual stage antigens due to the lack of MHC I proteins on red blood cells.

### 2.5. Design and implementation of T cell epitope down-selection tool

**Figure 4.**
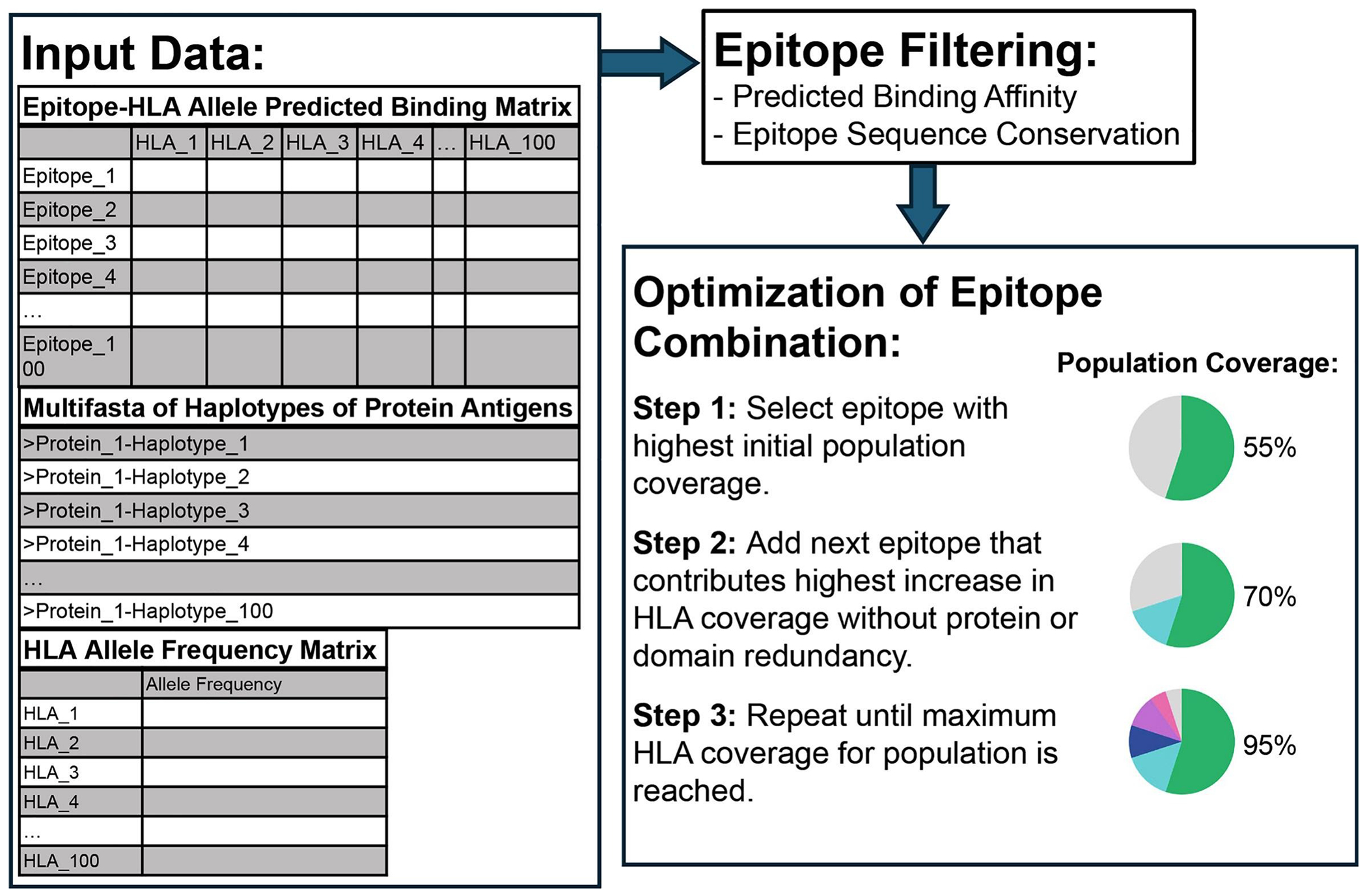
Workflow of T Cell Epitope Nomination (TEpiNom) algorithm. Input data includes epitope-HLA allele predicted binding matrix, multifasta of sequence for each protein antigen haplotype, and HLA allele frequency matrix. After epitope filtering using user-defined thresholds for median predicted binding affinity and epitope sequence conservation, the tool optimizes a combination of epitopes to maximize HLA coverage in the population, while minimizing target redundancy.

To streamline T cell epitope down-selection, we developed a Python-based computational tool, the T cell Epitope Nomination Tool (TEpi-Nom; Patent Pending; https://github.com/alexlaurenson/epiweight), which integrates parasite protein sequence datasets, predicted epitope-HLA binding data, and HLA allele frequency datasets to down-select for or nominate a list of candidate T cell epitopes (Fig. 4) [91–93]. TEpiNom first filters epitopes based on two criteria: (1) conservation across protein sequence datasets, and (2) predicted HLA binding affinity. Conservation (*C*) was calculated using the proportion of the protein sequence dataset in which an epitope is 100% conserved, specifically with the following formula: $C=\frac{N_{\mathrm{epitope}}}{N_{\mathrm{total}}}$, where *N_epitope_* is the number of sample sequences for the respective protein that contain the exact epitope sequence and *N_total_* is the total number of sample sequences for that protein. Predicted HLA binding affinity ranks derived from NetMHCpan-4.1 and NetMHCIIpan-4.1, reflect the predicted strength of epitope binding to specific HLA alleles with lower percentage ranks indicate stronger predicted binding. TEpiNom was used to apply cutoffs of epitope sequence conservation greater than 95%, to prioritize epitopes that are conserved across parasite sequences to a high degree, and median binding affinity ranks below 10%, to retain epitopes predicted to interact with at least some degree of medium or strong affinity across a broad set of MHC I or II alleles, as selection for allele-specific strong binding would be included in the optimization step.

After filtering for epitope-HLA binding strength and epitope sequence conservation, the tool applies integer linear programming to down-select epitopes based on specific goals. TEpiNom’s primary objective is to maximize predicted HLA allele population coverage based on each allele’s phenotypic frequency (*q*), derived from allele frequency (*f*) using the formula: *q* = 2*f* – *f*^2^. Phenotypic frequency represents the probability that an individual carries at least one copy of a given HLA allele and therefore can theoretically present epitopes restricted by that allele. For each epitope, TEpiNom identifies all HLA alleles for which the predicted binding affinity rank falls below a 0.5% for MHC I epitopes and 1% for MHC II epitopes, thresholds defined by NetMHCpan-4.1 and NetMHCIIpan-4.1 as covered by that epitope [33]. Then, within each HLA genetic locus (HLA-A, HLA–B, HLA-C for MHC class I; DRB1, HLA-DPA1, HLA-DQA1 for MHC class II), TEpiNom aggregates the phenotypic frequencies of all HLA alleles covered by the candidate epitope or epitope set and computes intra-locus coverage using a formula described by the IEDB (Immune Epitope Database) [94]: ${Coverage}_{\mathrm{int}ra–locus}=1-\overset{k}{\underset{i=1}{\prod }}\left(1-q_{i}\right)$ where *q_i_* is the phenotypic frequency of each covered HLA *i*=1 allele within that locus and *k* is the number of covered alleles. The *Coverage_intra–locus_* reflects the probability that an individual carries at least one allele from that locus capable of presenting the selected epitope (s). To calculate population coverage across all HLA loci for either MHC I or II, TEpiNom uses the following formula [94] starting with coverage within the first locus and iteratively updating the inter-locus coverage (*Coverage_across loci_*) with each remaining loci coverage.

$${Coverage}_{across\;loci}=1\times {Coverage}_{locus\_1}$$

$${Coverage}_{across\;loci}+=\left(1-{Coverage}_{across\;loci}\right)\times {Coverage}_{locus\_2}$$

$${Coverage}_{across\;loci}+=\left(1-{\mathrm{Coverage}}_{\mathrm{across loci}}\right)\times {Coverage}_{locus\_3}$$

TEpiNom uses these population coverage calculations to evaluate all candidate epitopes and incrementally construct larger epitope sets, identifying the best performing combination at each set size until population coverage reaches 100% or until no further increase is possible. TEpiNom also identifies epitope-dense regions by mapping filtered epitopes to their source proteins and amino acid coordinates. For each protein, the tool scans genomic windows and calculates the combined population coverage of all epitopes within that interval. Regions that achieve high coverage over the defined genomic window size are prioritized as optimal targets for construct design. In this research, TEpiNom’s epitope-dense region optimization was run using genomic window sizes of 50, 100, 150, and 200aa. For comparison to the calculated population coverage achieved by RTS,S, the vaccine’s 189aa CSP genomic region (199-387aa) was included in TEpiNom’s genomic region coverage model [95].

To accommodate additional down-selection goals, TEpiNom accepts input modifiers that adjust the objective function during optimization. One modifier alters solver behavior in epitope combination selection to increase the weight of epitopes drawn from different proteins, improving antigenic breadth, and potentially reducing immune pressure on any single protein. TEpiNom can also assign higher priority to specific HLA alleles associated with elevated risk or severe clinical outcomes.

### 2.6. Literature search for clinically associated HLA types

To contextualize the clinical relevance of HLA-restricted T cell epitopes, we conducted a structured literature review to identify HLA alleles associated with parasitemia, uncomplicated malaria, or severe malaria. Searches were performed using PubMed with combinations of the following keywords: “*Plasmodium falciparum*,” “malaria,” “HLA,” “parasitemia,” “uncomplicated malaria,” “severe malaria,” and “cerebral malaria,” to screen titles, abstracts, and full texts. Studies were included if they provided HLA allele or allele group genotyping, clearly defined clinical endpoints, and reported statistically significant associations between HLA alleles or allele groups and parasitemia, uncomplicated malaria, severe malaria, cerebral malaria, or severe malarial anemia. We excluded case reports, *in vitro* or animal model studies, and analyses without well-defined clinical outcomes. For each included study, we extracted the reported HLA allele or allele group, direction of association (protective or non-protective association), and associated clinical endpoint (Table 1). HLA alleles with significant positive associations with severe malaria outcomes were included in epitope combination and epitope-dense region optimization as “high-risk HLA alleles” for prioritized epitope coverage.

### 2.7. Performance benchmarking of TEpiNom epitope combination optimization of predicted population coverage

To evaluate TEpiNom performance relative to the greedy optimization epitope selection strategy used in PopCover-2.0 [38], we generated a synthetic benchmarking framework that systematically varied objective difficulty and combinatorial complexity across 100 simulation tiers with increasing difficulty. For each simulation replicate, an epitope-HLA binding matrix and HLA allele frequency dataset were constructed. In lower tiers, programmed epitope sets exhibited clean separability of predicted allele coverage, yielding an easily identifiable and near optimal solution. In higher tiers, simulated datasets incorporated progressively greater levels of partially overlapping coverage and redundancy. TEpiNom and PopCover-2.0 used identical binding matrices and allele frequency distributions within each simulation to ensure fair comparison. For each run, both models attempted to find the smallest epitope set achieving maximal population coverage, allowing assessment of solution compactness and coverage efficiency. For each difficulty tier, we performed 100 independent simulations, and model performance was summarized as the predicted population coverage achieved by each method using the smallest number of epitopes. We calculated the median performance of each model within each tier and derived the performance delta (TEpiNom minus PopCover-2.0) as a function of increasing tier complexity. Statistical significance of performance differences between TEpiNom and PopCover-2.0 within each tier was assessed using the Wilcoxon signed-rank test.

### 2.8. T cell epitope prediction and analysis of PfNF54 CSP against RTS,S vaccine efficacy

To evaluate the T cell epitope landscape within the PfNF54 CSP, we performed MHC I and II epitope predictions using the same NetMHCpan-4.1 and NetMHCIIpan-4.1 parameters described above *(Methods: Prediction of T cell epitopes)* for HLA alleles significantly associated with either RTS,S-mediated protection (HLA-A*01 and HLA-B*08 for MHC I; HLA-DRB1*15 and HLA-DRB1*16 for MHCII) or lack of protection (HLA-A*03 and HLA-B*53 for MHC I; HLA-DRB1*07 for MHCII) against controlled human malaria infection [7]. Binding of each epitope to each allele was categorized using NetMHCpan-4.1/NetMHCIIpan-4.1 rank-based thresholds: strong binding (<0.5% for MHC I, <1% for MHC II), weak binding (<2% for MHC I, <10% for MHC II), or non-binding [33]. We conducted a per-allele analysis in which the predicted epitope binding calls were enumerated separately for each allele. For each region analyzed, the per-allele distributions of strong, weak, and nonbinding epitope counts were compared between protective *versus* non-protective HLA allele groups. Statistical testing was performed using the Mann-Whitney *U* test to compare allele-level distributions between groups. These analyses were repeated within the well-described T cell epitope regions, Th2R (311-327aa) and Th3R (341-364aa), within CSP associated with vaccine efficacy [96–98]. For each region, per-allele binding classifications (strong, weak, none) were tabulated, group-stratified by protection status, and tested for differences using the same nonparametric framework.

## 3. Results

### 3.1. Sequence diversity within candidate antigens

**Figure 5:**
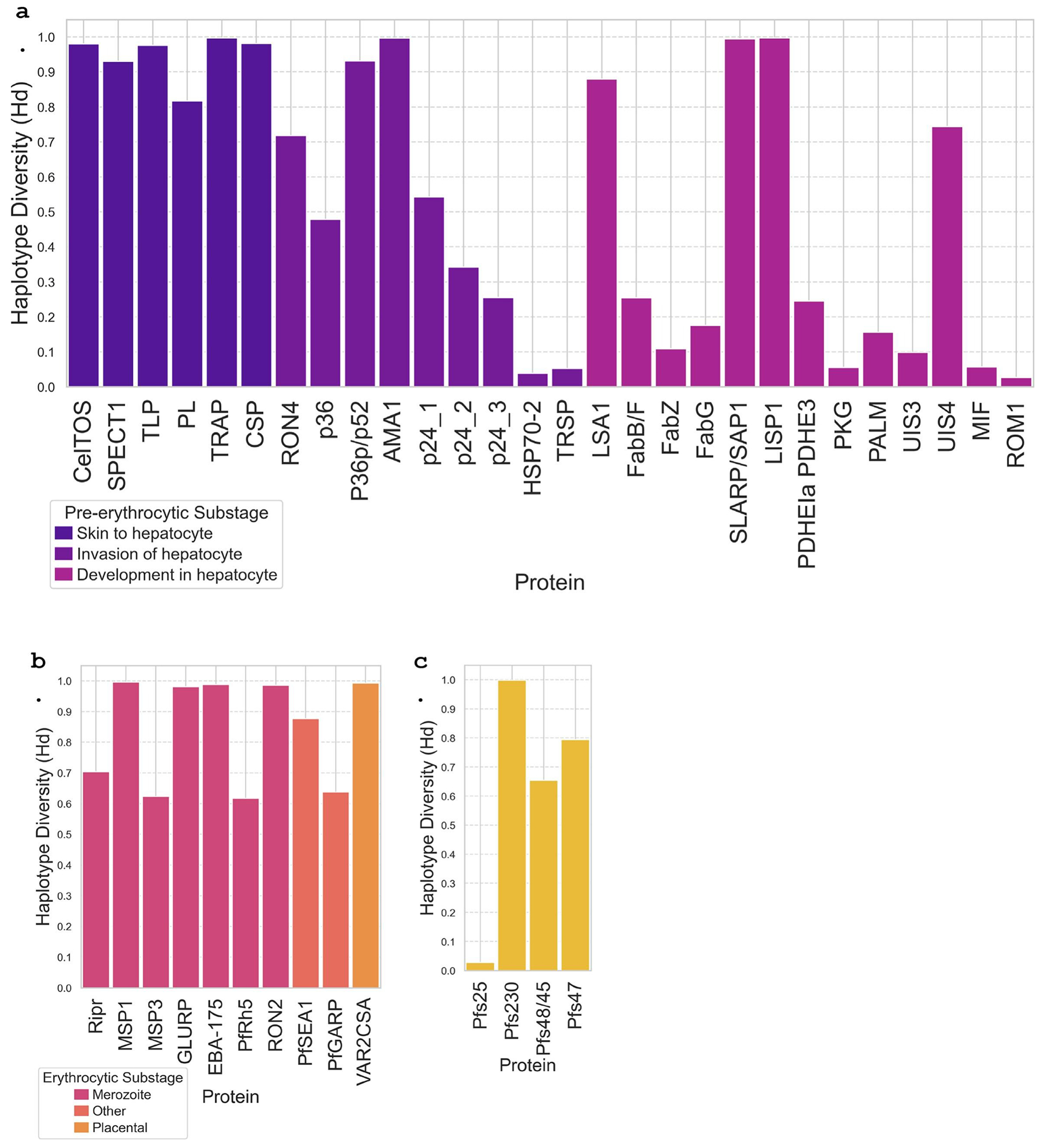
Haplotype diversity for malaria vaccine candidate proteins across different life cycle stages. Box plots show the distribution of haplotype diversity values for protein candidates within the **a. Pre-erythrocytic stage**, **b. Erythrocytic stage**, and **c. Sexual stage**, with sub-stages of protein function labeled. Haplotype diversity ranges from 0.00 to 1.00 and reflects the probability that two randomly chosen haplotypes from the population are different. Higher values indicate sequence diversity, and values closer to 0.00 reflect sequence conservation.

Haplotype diversity analysis across the protein sequence datasets (Supplementary Table 2) revealed varying levels of sequence diversity within each protein antigen (Fig. 5). In the pre-erythrocytic stage, several proteins such as TRAP (0.99), AMA1 (0.99), CSP (0.98), and CelTOS (0.98) displayed high haplotype diversity values, indicating substantial sequence variation among circulating strains (Fig. 5a). Conversely, multiple pre-erythrocytic stage proteins, including HSP-70 (0.04), TRSP (0.05), PKG (0.06), and ROM1 (0.03), exhibited low haplotype diversity, suggesting a high degree of sequence conservation. Erythrocytic stage proteins also spanned a wide range of diversity, with MSP1 (0.99), GLURP (0.98), and EBA-157 (0.99) among the most diverse, and PfRh5 (0.62) and PfSEA1 (0.88) showed moderate diversity (Fig. 5b). In the sexual stage, Pfs230 (0.99) was highly diverse, whereas Pfs25 (0.03) and Pfs48/45 (0.65) were more conserved (Fig. 5c). These results demonstrate that many candidate proteins are highly polymorphic, and others show strong conservation, supporting inclusion of conserved antigens and epitopes in vaccine formulations.

### 3.2. Prediction and filtering of T cell epitopes within candidate proteins

To identify T cell epitopes within the protein antigens, we used the NetMHCpan-4.1 and NetMHCIIpan-4.0 tools to predict epitopes and their binding affinity ranks to MHC I and II proteins. Through this method, we predicted 244,036 unique MHC I and 164,034 unique MHC II epitopes, all with different predicted binding affinity ranks to the input HLA alleles. We then used the TEpiNom workflow to filter epitopes based on their median binding affinity rank (<10%) to the HLA alleles and epitope sequence conservation (>95%). Predicted epitopes were retained only if they met both criteria.

After applying these filter thresholds, we identified 2265 MHC I and 1992 MHC II candidate epitopes across malaria vaccine candidate proteins and characterized the retention rate per protein, or the proportion of candidate epitopes that remained after filtering (Supplementary Table 3).

**Figure 6:**
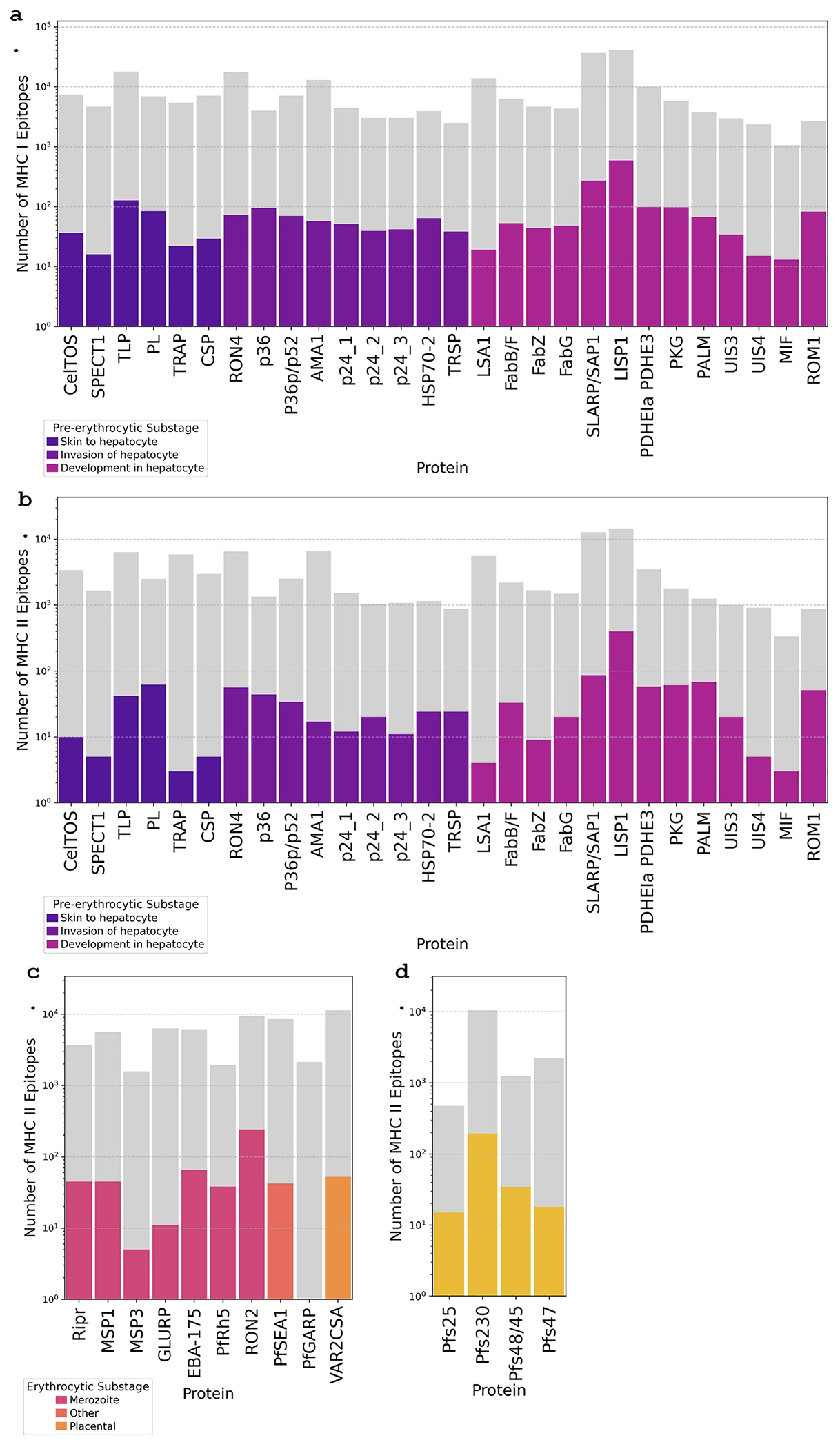
Predicted and retained T cell epitopes across malaria vaccine candidate proteins. Number of predicted MHC I and MHC II epitopes before and after applying conservation and binding affinity filters (<10% median binding rank, >95% conservation). Gray shading indicates epitope counts prior to filters, and colored shading indicates after. Data is plotted on a logarithmic scale to show resolution and categorized by malaria life cycle stage (**a. Pre-erythrocytic MHC I epitopes, b. Pre-erythrocytic MHC II epitopes, c. Erythrocytic MHC II epitopes, and d. Sexual stage MHC II epitopes**) and substage with epitope counts shown for each protein after filtration steps. MHC I epitope predictions performed exclusively for pre-erythrocytic stage proteins.

For MHC I epitope predictions, conducted exclusively for pre- erythrocytic antigens, multiple highly conserved and promiscuous epitopes were identified across all candidates (Fig. 6a; Supplementary Table 3). ROM1, p36, and PALM had the highest MHC I epitope retention rates (3.08% and2.37%, and 1.80%, respectively), suggesting these proteins contain regions with high sequence conservation and predicted epitope-HLA interactions. SLARP/SAP1 and LISP1 had the highest absolute numbers of retained MHC I epitopes, with 584 and 270 epitopes, respectively, indicating strong potential as T cell targets.

For MHC II epitopes, critical for eliciting CD4+ T cell responses, the number and percentage of retained epitopes were similar to epitopes predicted to bind MHC I. Among pre-erythrocytic stage proteins, ROM1 (5.88%), PALM (5.40%), PKG (3.39%), and p36 (3.26%) had the highest retention percentages, suggesting their potential as strong CD4+ T cell inducers (Fig. 6b; Supplementary Table 3). The pre-erythrocytic stage LISP1 had the highest absolute number of retained MHC II epitopes (400), followed by SLARP/SAP1 (86). Within erythrocytic antigens, RON2 retained the most MHC II epitopes (241, 2.56%), followed by PfRh5 (1.98%) and EBA-175 (1.09%) (Fig. 6c; Supplementary Table 2). In contrast, GLURP, MSP3, and PfGARP had extremely low retention rates (<0.5%), indicating either greater sequence variability or weaker predicted binding affinities. For sexual stage antigens, retention rates were highest for Pfs25 (3.15%), Pfs48/45 (2.74%), and Pfs230 (1.84%), although low compared with those of pre-erythrocytic and erythrocytic stage antigens (Fig. 6d; Supplementary Table 3).

Importantly, liver stage antigens ROM1, p36, and PALM had high epitope retention rates for both MHC I and MHC II, suggesting these proteins contain conserved epitope repertoires with broad predicted HLA presentation to both CD8+ and CD4+ T cells.

### 3.3. Optimization of T cell epitope combinations for predicted population-level and high-risk HLA allele coverage

After initial epitope filtering, we used TEpiNom to optimize MHC I and II epitope combinations that maximize predicted HLA population coverage while minimizing redundancy across parasite proteins. TEpi-Nom also prioritized predicted coverage of high-risk HLA alleles associated with severe clinical malarial outcomes (Supplementary Table 4a–b).

**Figure 7.**
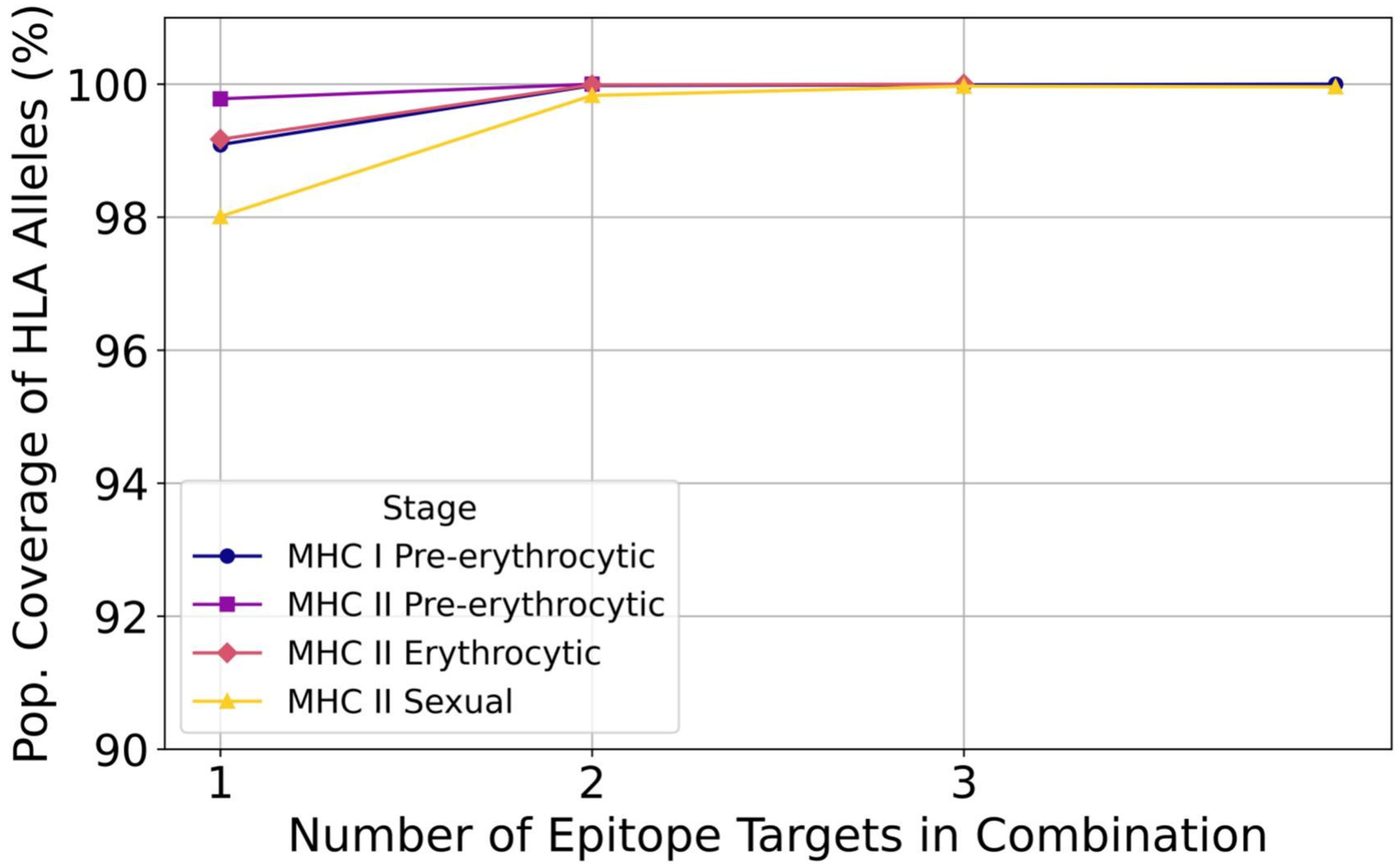
Population coverage of optimized epitope combinations. Population coverage (%) of HLA alleles for MHC I and MHC II epitope combinations of increasing number within each malaria life cycle stage. Epitope combinations were selected from filtered data sets based on conservation and binding affinity criteria. The number of epitopes within a set increased until 100% inter-locus population coverage was reached for that MHC I or II and life stage category.

For MHC I pre-erythrocytic stage epitopes, a single epitope from p36 (MAYNIWEEY) reached 99.09% inter-locus coverage, and the highest-performing combination, FabZ (LPHRYPFLL), FabG (RTNLNSLFY), p36 (YIMANFHNV), and PKG (YEFICGPLPF), achieved 100% inter-locus MHC I coverage with high degrees of epitope sequence conservation (Fig. 7; Supplementary Table 3a). All combinations provided coverage of high-risk alleles A29:02 and A66:02, and the final combination increased coverage to include A*30:01, but did not cover high-risk HLA allele A*33:01 (Supplementary Table 4a).

Optimization of epitope combinations for MHC II pre-erythrocytic stage epitopes identified a single epitope from PL (RKDFISFRITKLIKL) that produced 99.78% inter-locus MHC II coverage, and the top two-epitope combination PALM (RKDFISFRITKLIKL) and PL (MKIIIASSAAVAVLA) reached 100% inter-locus coverage (Fig. 7; Supplementary Table 4b). Both combinations provided full coverage of the high-risk DRB1*04 allele group (9/9 alleles) (Supplementary Table 3b).

Within MHC II erythrocytic stage epitopes, optimization yielded an EBA-175 epitope (QNKYVPINAVRVSRI) that achieved 99.17% inter-locus coverage. Adding the RON2 epitope (VWKVISSFALHHLKN) increased this to 99.99%. The full three-epitope combination with MSP1 (KKRKYFLDVLESDLM) reached 100% coverage, with all epitopes showing high degrees of conservation (Fig. 7; Supplementary Table 3c). All erythrocytic combinations robustly covered high-risk DRB1*04 allele group, with the two- and three-epitope sets covering 9/9 DRB1*04 alleles (Supplementary Table 4c).

For MHC II epitopes within sexual stage proteins, coverage progressively increased with the addition of epitopes from Pfs230, Pfs48/45, Pfs47, and Pfs25. A single Pfs48/45 epitope (HSYFIYDKIRLIIPK) provided 98.01% coverage, and the highest-performing combination, Pfs25 (FLFIQLSIKYNNAKV), Pfs230 (IRSVLQSGALPSVGV), Pfs47 (KYAINSSFSDFYLKI), and Pfs48/45 (HSYFIYDKIRLIIPK), achieved 99.96% inter-locus coverage with uniformly high conservation (Fig. 7; Supplementary Table 4d).

### 3.4. Performance benchmarking of TEpiNom epitope combination optimization of predicted population coverage

**Figure 8.**
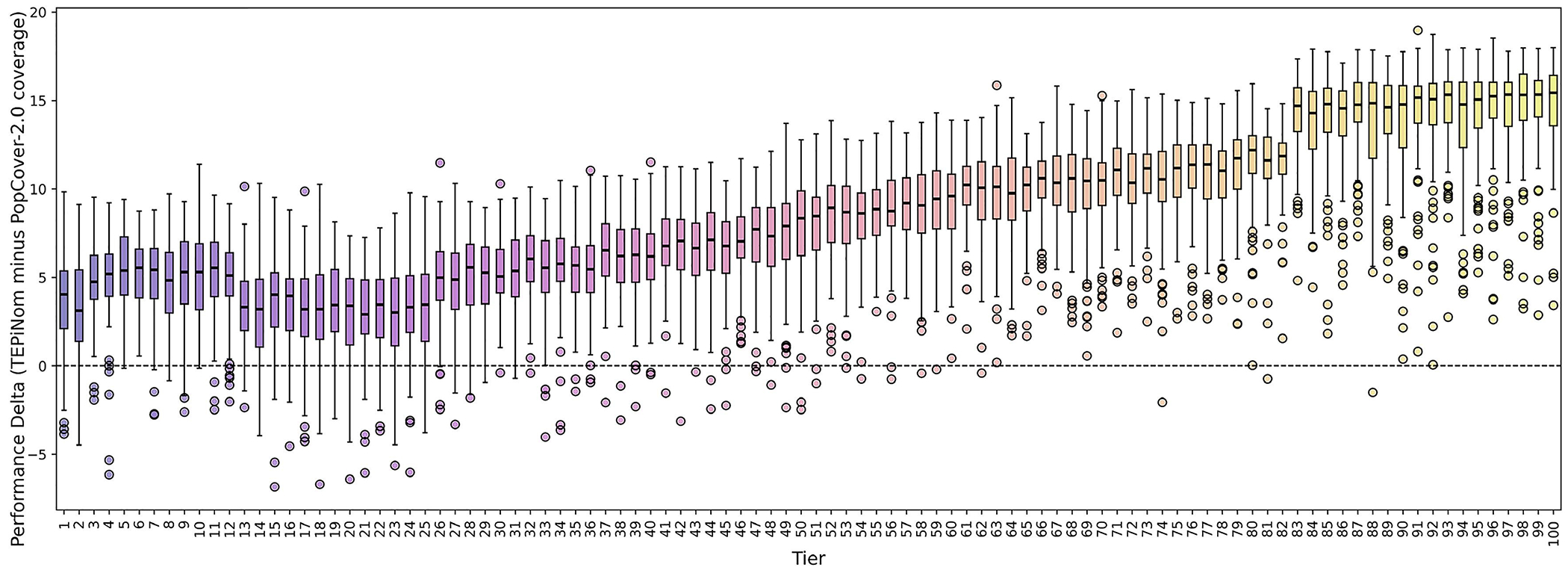
Median performance delta between TEpiNom and PopCover-2.0 across simulation tiers. The performance difference was calculated as the median population coverage achieved by TEpiNom minus the median population coverage achieved by PopCover-2.0, using equivalently sized epitope solutions for each simulation dataset. Each point represents the median performance delta within a difficulty tier, each tier was evaluated using 100 independent simulations, and the curve shows the trend in relative performance across 100 tiers of increasing combinatorial complexity. Statistical significance of the paired comparison within each tier was evaluated using the Wilcoxon signed-rank test, with all tiers showing p-values less than 0.001.

Across all tiers, TEpiNom’s integer linear programming method consistently identified epitope sets achieving better predicted population coverage than PopCover-2.0’s greedy optimization method using the same simulated input data (Fig. 8). The median inter-locus population coverage advantage of TEpiNom over PopCover-2.0 in low complexity tiers ranged from approximately 3–6%. The median performance difference commensurately increased with tier difficulty, reaching values of approximately 10–12% in midrange tiers and 15% in the highest tiers (Fig. 8). TEpiNom performance remained superior to PopCover-2.0 throughout the full tier spectrum (Fig. 8).

### 3.5. Optimization of T cell epitope-dense regions for HLA and parasite sequence coverage

For MHC I pre-erythrocytic stage proteins, 497 distinct protein regions each achieved 100% inter-locus population coverage in only 50aa (data not shown). Among the top-hit regions within the 50aa window size, regions from TRSP, p36, ROM1, and LISP1 provided 100% inter-locus coverage while also covering three of the four high-risk HLA alleles, A*29:02, A*30:01, and A*66:02 (Supplementary Table 5a). When the window size was increased to 100aa, regions from p24_1, RON4, and LISP1 each provided 100% inter-locus coverage and covered the same high-risk allele set, with multiple LISP1 regions also covering high-risk allele A*33:01, but sacrificing the coverage the A*30:01 (Supplementary Table 5a). Notably, no regions within any pre-erythrocytic stage proteins achieved coverage of high-risk alleles A*30:01 and A*33:01 simultaneously.

Optimization of pre-erythrocytic MHC II epitope-dense regions identified numerous regions with 100% inter-locus population coverage, with 1047 distinct regions of 50aa genomic window size (data not shown). The highest-performing 50aa regions included ones from PALM, TLP, PL, SLARP or SAP1, and LISP1, which all fully covered the HLA-DRB1*04 high-risk allele group and achieved 100% inter-locus coverage (Supplementary Table 5b). For MHC II erythrocytic stage proteins, 517 50aa regions were identified that reached 100% inter-locus coverage (data not shown), with regions from PfRh5, MSP1, and RON2 among the top hits that also fully covered the DRB1*04 allele group (Supplementary Table 5c). Within sexual stage proteins, 202 MHC II epitope-dense regions at the 50aa window size reached 100% inter-locus coverage (data not shown), including regions from antigens Pfs230 and Pfs48/45 (Supplementary Table 5d).

TEpiNom’s predicted regional population coverage model was applied to the CSP region included in the RTS,S vaccine to calculate population coverage for the complete vaccine sequence, and to compute coverage and sequence conservation metrics for individual epitopes. For MHC I, the RTS,S CSP region reached 82.48% HLA-A coverage, 96.31% HLA-B coverage, 96.01% HLA-C coverage, and 99.97% inter-locus coverage (Supplementary Table 6a). Although the inter-locus value was high, the individual epitope contributors exhibited low sequence conservation for many peptides responsible for HLA-A and HLA-B coverage. The dominant contributor to overall coverage was YLNKIQNSL, which provided 93.55% inter-locus coverage but had an epitope sequence conservation value of 0.130 (Supplementary Table 6a). Multiple epitopes from the C-terminus region, including STEWSPCSV, TEWSPCSVT, and TEWSPCSVTC, had uniformly high sequence conservation but contributed only modest coverage at single loci (Supplementary Table 6a). For MHC II, the RTS,S CSP region produced substantially lower coverage relative to optimized pre-erythrocytic, erythrocytic, and sexual stage regions. The RTS,S CSP segment reached 77.56% DRB1 coverage, 0% DPA1 to DPB1 coverage, 15.29% DQA1 to DQB1 coverage, and 80.99% inter-locus coverage (Supplementary Table 6b). The highest performing MHC II epitopes from the region, including HIKEYLNKIQNSLST, IKEYLNKIQNSLSTE, KEYLNKIQNSLSTEW, and EYLNKIQNSLSTEWS, covered 25–30% of DRB1 each, but showed zero coverage at the DPA1 to DPB1 locus, and only 0.61% coverage at the DQA1 to DQB1 locus (Supplementary Table 6b). These epitopes also displayed low conservation values of 0.127 to 0.130. Additional C-terminus epitopes such as GIQVRIKPGSANKPK and IQVRIKPGSANKPKD provided only 3.32% inter-locus coverage with conservation values below 0.90 (Supplementary Table 6b).

### 3.6. Predicted T cell epitopes and binding profiles within RTS,S CSP Region

**Figure 9.**
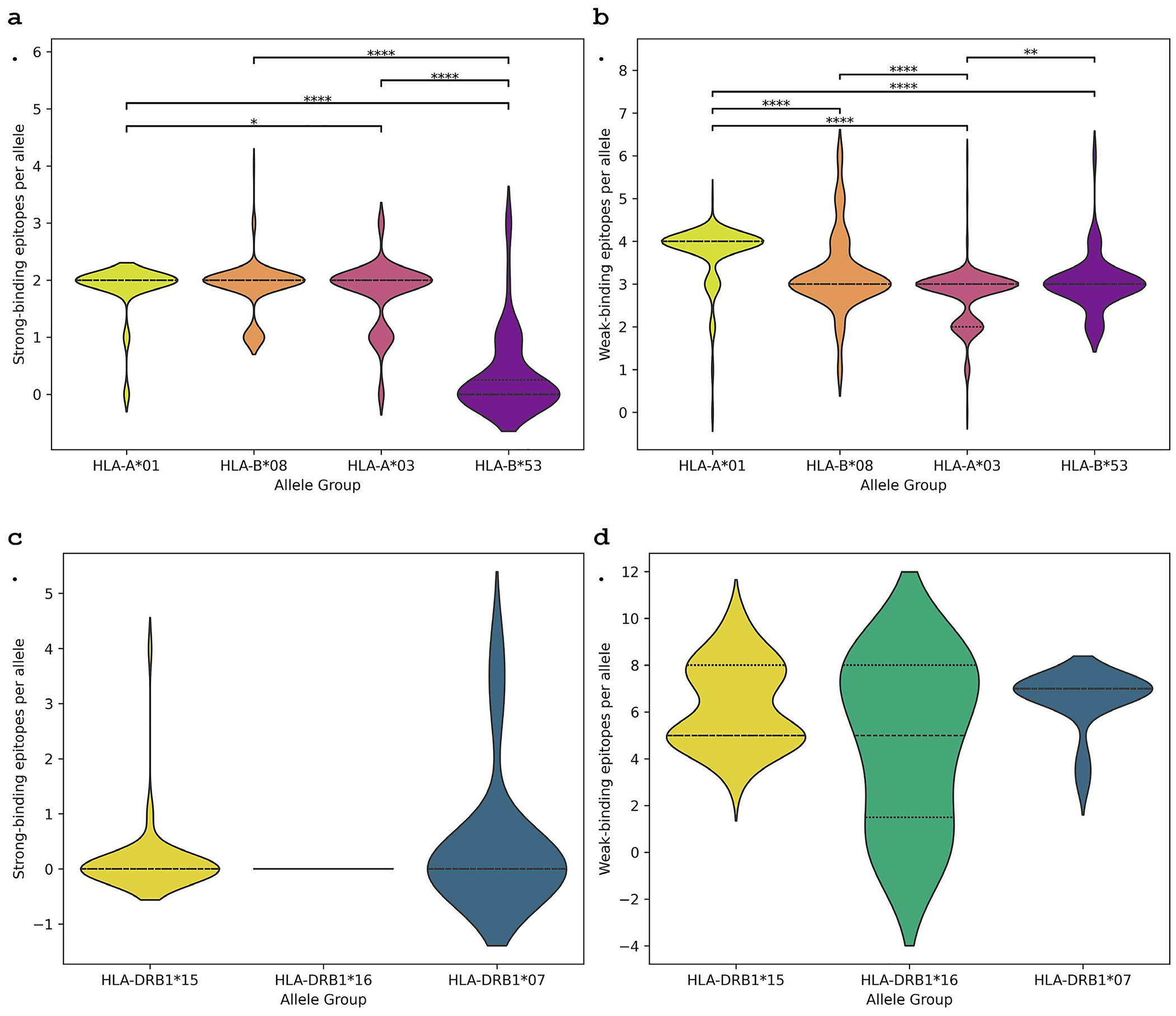
T cell epitope predictions across the full PfNF54 CSP region included in RTS,S. Violin plots depicting the distribution of predicted T cell epitopes for protective and non-protective HLA allele groups within the CSP region included in the RTS,S vaccine. Protective alleles for MHC I are HLA-A*01 and HLA-B*08, and for MHC II are HLA-DRB1*15 and HLA-DRB1*16; non-protective alleles for MHC I are HLA-A*03 and HLA-B*53, and for MHC II is HLA-DRB1*07. Each panel shows the number of predicted epitopes per HLA allele within each allele group: a. Number of strong-binding MHC I epitopes per allele, b. Number of weak-binding MHC I epitopes per allele, c. Number of strong-binding MHC II epitopes per allele, d. Number of weak-binding MHC II epitopes per allele. Strong and weak binding were defined according to NetMHCpan-4.1 and NetMHCIIpan-4.1 recommended rank thresholds (strong: <0.5% for MHC I, <1% for MHC II; weak: <2% for MHC I, <10% for MHC II). Statistical testing was performed using the Mann-Whitney U test to compare allele-level distributions between groups.

Analyses of predicted T cell epitopes within the CSP region included in RTS,S showed variations in the numbers of strong and weak epitope interactions per HLA allele across allele groups associated with either increased or decreased RTS,S-mediated protection against controlled human malaria infection (Fig. 9). Non-protective allele group B*53 consistently exhibited significantly lower predicted strong and weak epitope counts relative to other allele groups, including its non-protective counterpart A*03 (Fig. 9a,b). Protective allele groups A*01 and B*08 were predicted to present similar numbers of strong epitopes per allele group, with medians of 2 and mean counts of approximately 1.86 strong epitopes per group (Fig. 9a). Protective A*01 exhibited significantly higher weak epitope counts per allele than all other allele groups, and statistically significant higher strong epitope counts than non-protective A*03, although the effect size was small (Cliff’s delta = 0.077) (Fig. 9a,b). For MHC II epitopes within the RTS,S CSP region, strong-binding epitopes were generally rare across both protective (DRB1*15 and DRB1*16) and non-protective (DRB1*07) allele groups (Fig. 9c). Weak-binding epitope counts varied modestly and insignificantly (Fig. 9d).

**Figure 10.**
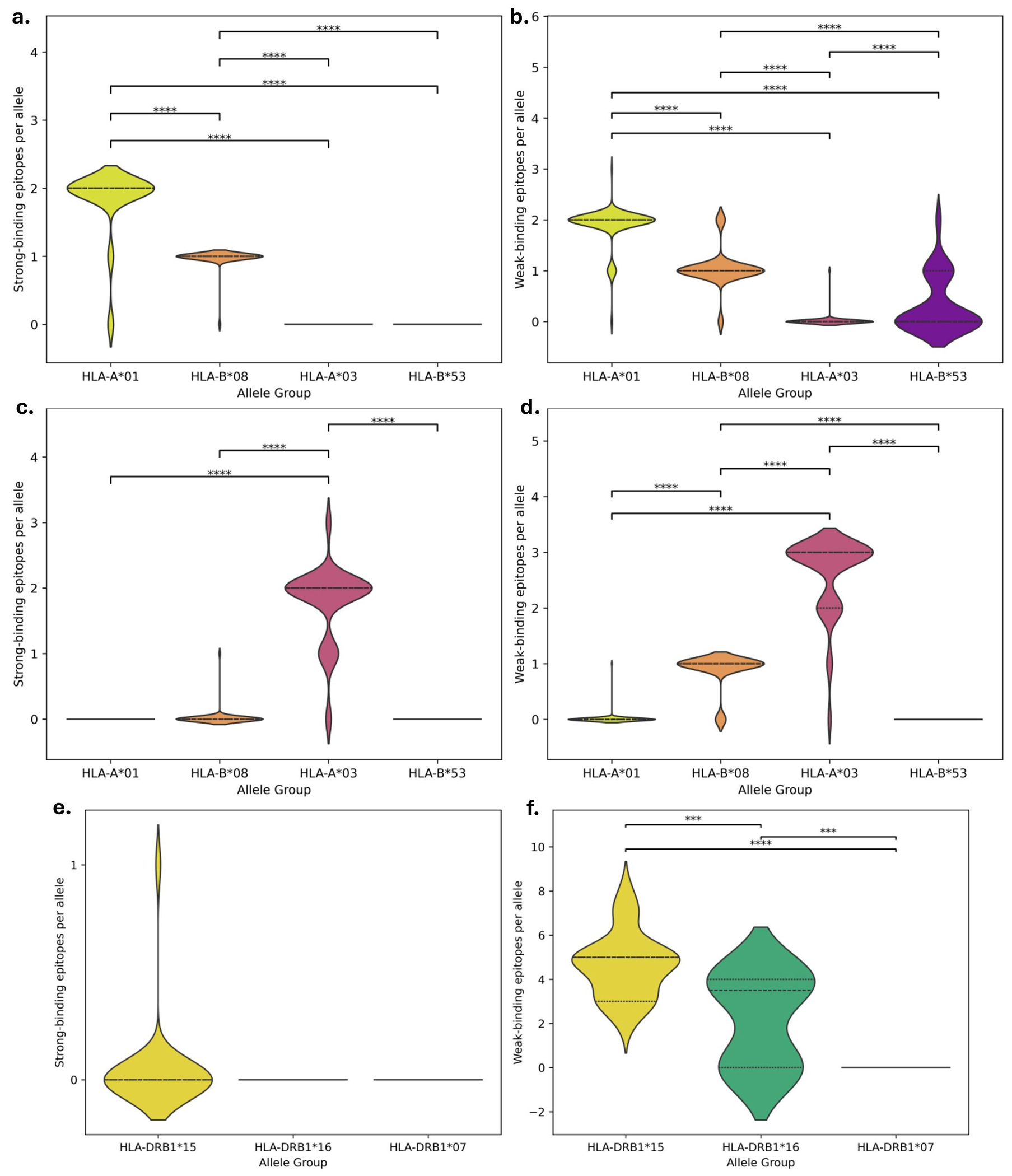
T cell epitope predictions within CSP Th2R and Th3R regions. Violin plots showing the number of predicted T cell epitopes per HLA allele within the Th2R and Th3R CSP motifs, stratified by protective versus non-protective HLA allele groups. Protective alleles for MHC I are HLA-A*01 and HLA-B*08, and for MHC II are HLA-DRB1*15 and HLA-DRB1*16; non-protective alleles for MHC I are HLA-A*03 and HLA-B*53, and for MHC II is HLA-DRB1*07. Panels display: a. Number of strong-binding MHC I epitopes per allele in Th2R, b. Number of weak-binding MHC I epitopes per allele in Th2R, c. Number of strong-binding MHC I epitopes per allele in Th3R, d. Number of weak-binding MHC I epitopes per allele in Th3R, e. Number of strong-binding MHC II epitopes per allele in Th2R, f. Number of weak-binding MHC II epitopes per allele in Th2R. Strong and weak binding were defined according to NetMHCpan-4.1 and NetMHCIIpan-4.1 recommended rank thresholds (strong: <0.5% for MHC I, <1% for MHC II; weak: <2% for MHC I, <10% for MHC II). Statistical testing was performed using the Mann-Whitney U test to compare allele-level distributions between groups.

Predicted T cell epitope binding intensity analyses focused specifically on the well-studied CSP Th2R and Th3R regions found variable responses across associated MHC I allele groups (Fig. 10). Within the Th2R region, protective A*01 exhibited the highest median strong epitope count per allele, followed by B*08, while non-protective A*03 and B*53 showed medians of 0 for strong epitope counts (Fig. 10a). Pairwise comparisons indicated that A*01 differed significantly from all non-protective groups and B*08 in strong epitope counts per allele (Fig. 10a). In the Th3R region, median strong epitope counts were generally low across all MHC I allele groups, with B*08 showing a median of 1 and all others at 0 (Fig. 10c). Weak epitope counts were low, with A*03 showing a median of 3, A*01 and B*08 medians of 1, and B*53 near 0 (Fig. 10d). Predicted MHC II epitope binding remained sparse across allele groups within Th2R and Th3R (Fig. 10). In Th2R for MHC I, strong epitope counts were essentially zero for all groups (Fig. 10e). Weak epitope counts were significantly higher in protective DRB1*15 and DRB1*16 compared to non-protective DRB1*07, with medians of 5, 3.5, and 0, respectively (Fig. 10f). Within Th3R, strong and weak MHC II epitope counts were zero across all DRB1 allele groups, with no statistically significant differences observed.

## 4. Discussion

This study presents TEpiNom, a computational framework for rational T cell epitope down-selection in vaccine design, integrating pathogen diversity and regional HLA allele frequency to prioritize conserved epitopes with predicted promiscuous binding profiles and broad population coverage. Applying this approach to *Pf*, we identified predicted MHC I and II epitopes across multiple parasite life stages that could inform strategies to address antigenic diversity and HLA restriction, two major hurdles in malaria vaccine efficacy [5–7]. In addition to curated T cell epitope datasets, TEpiNom generates highly conserved epitope combinations designed to maximize predicted inter-locus HLA coverage under strict constraints to minimize target redundancy, and epitope-dense genomic regions that achieve broad predicted HLA coverage with sustained sequence conservation.

Our findings align with previous studies documenting HLA-restricted immune responses to *Pf* antigens and demonstrate that an epitope-based vaccine approach can prioritize conserved regions located across pre- erythrocytic, erythrocytic, and sexual stage proteins for experimental evaluation [7,17]. This work expands on earlier efforts validating CD4+ T cell epitopes in erythrocytic stage antigens, by broadening predictions and down-selection to thousands of isolate sequences of 42 candidate malaria vaccine protein antigens against hundreds HLA alleles common in endemic regions [36]. Using variant *Pf* sequences rather than a single laboratory strain enhances relevance to circulating strains causing disease in endemic settings. Incorporating HLA allele frequencies from diverse sub-Saharan African populations further increases the likelihood that prioritized epitopes are immunologically relevant to populations at highest malaria risk. Collectively, TEpiNom provides a methodological framework that supports rational selection and experimental validation of vaccine targets, with the downstream objective to elicit robust immunity across genetically diverse populations and overcoming HLA restriction observed with RTS,S [7].

Analyses of predicted T cell epitopes in the RTS,S CSP segment underscore the value of population-specific epitope binding predictions. HLA B*53 displayed the weakest predicted epitope presentation across the RTS,S CSP region, with significantly fewer strong and weak MHC I binders relative to other allele groups, consistent with previous reports linking B*53 with reduced RTS,S-mediated protection [7]. Predicted MHC II binding was sparse across DRB1 groups, with no differences in binding intensity, compatible with the relatively weak association between DRB1*15/*16 and RTS,S-mediated protection [7]. This aligns with clinical evidence that RTS,S can generate protective CD4+ T cell responses [99], though often with variable magnitude and frequency, especially in genetically heterogeneous populations, suggesting that inconsistent CD4+ T cell priming may stem from MHC II HLA haplotype and the resulting underlying differences in epitope presentation [99–101]. Together, these findings reinforce that differences in epitope presentation across HLA backgrounds likely contribute to variable RTS,S efficacy and underscore the utility of population-level epitope prediction frameworks for candidate prioritization.

To further assess TEpiNom’s performance, we compared down-selected epitopes, filtered for conservation and predicted binding promiscuity, against IEDB-catalogued epitopes supported by positive T cell assays [102]. TEpiNom and IEDB identified the same 145 MHC I and 21 MHC II epitopes across 10 antigens (Supplementary Table 8), demonstrating TEpiNom’s concordance with experimentally validated epitopes and supporting its ability to rank both known and previously undescribed candidates for downstream validation.

Several tools support components of T cell epitope down-selection [37,38,94,103], but most address isolated steps. IEDB’s TepiTool integrates T cell epitope prediction with filtering for HLA allele binding affinity conservation [37], while another IEDB tool analyzes population coverage analysis but does not optimize epitope combinations [94]. In contrast, TEpiNom analyzes large epitope datasets, down-selects for conserved and broadly-binding epitopes, and identifies epitope combinations that maximize HLA allele coverage. PopCover-2.0 is a similar tool that incorporates epitope conservation and immune coverage [38] but may select for epitopes from the same antigen or that overlap in sequence, increasing susceptibility to immune escape. Conversely, TEpiNom avoids such redundancy, promoting breadth of antigen targeting. Benchmarking on simulated datasets found that TEpiNom’s integer linear programming consistently outperformed PopCover-2.0’s greedy algorithm across all complexity tiers in achieving maximal population coverage with the same number of epitopes. These features position TEpiNom as a substantial methodologic advance, providing an integrated framework for epitope prioritization, HLA coverage optimization, and redundancy-aware down-selection.

Despite these advantages, limitations remain. Although we evaluated 42 well-characterized Pf proteins across multiple life stages, this restricted list may exclude relevant targets. Future work will expand the antigen panel. The framework also depends on the accuracy of computational epitope-HLA binding predictions, which can include false positives or biases [33]. We used a single prediction algorithm because NetMHCpan and NetMHCIIpan are currently the only leading T cell epitope prediction tools supporting the full range of regional HLA alleles from sub-Saharan Africa. As additional tools expand allele coverage, consensus-based strategies will be incorporated. Constraints on peptide lengths, fixed for computational efficiency, may exclude legitimate epitopes, especially for MHC II, which tolerates substantial length variation. Future versions will evaluate variable length predictions as storage and computing constraints are relaxed. HLA allele frequency data remain incomplete in many malaria-endemic regions, meaning population coverage estimates reflect available data and may incompletely capture local HLA structure [87]. Although geographic specificity enhances relevance for endemic regions, it also limits generalizability. TEpiNom will be updated as new sub-Saharan African and geographically diverse HLA datasets become available. It is also important to note that TEpiNom predicts population-level epitope presentation likelihood but does not estimate vaccine efficacy or protective immunity, which depends on multiple biologic factors [104]. The framework prioritizes epitopes for experimental evaluation and does not infer protective responses.

Practical constraints also shape translation of epitope-based predictions into vaccine constructs. Multi-epitope formulations require optimized delivery platforms, potent adjuvants, and strategies to mitigate immunodominance [17]. Despite these limitations, successful multivalent vaccines, such as those targeting *Streptococcus pneumoniae* that protect against more than 20 serotypes [105], demonstrate that these challenges can be overcome.

Translating TEpiNom’s outputs into testable vaccine constructs begins with validating predicted epitopes using *in vitro* HLA binding or MHC stabilization assays, followed by *ex vivo* T cell ELISpot and intracellular cytokine staining studies employing HLA-typed donor cells from endemic regions [106]. Validated epitopes can then be assembled into an optimized vaccine construct with appropriate linkers and evaluated in HLA-expressing preclinical models to refine immunogenicity and assess functional responses [106]. This pipeline links computational predictions to experimental vaccine design

Beyond malaria, TEpiNom’s down-selection strategy is broadly applicable to pathogens with sequence diversity, complex life cycles, or with strong HLA restriction, including HIV, dengue, and influenza [107–109]. Its ability to rapidly prioritize conserved, promiscuously binding epitopes also supports pandemic preparedness by accelerating the early stages of antigen identification for emerging pathogens. Future development will focus on expanding pathogen-specific inputs and maintaining updated, region-specific HLA datasets.

This study demonstrates the feasibility of integrating pathogen sequence diversity, endemic HLA frequencies, and epitope presentation predictions to guide next-generation malaria vaccine development. Advancing computational predictions requires laboratory validation to confirm epitope immunogenicity using MHC stabilization assays, T cell stimulation studies, and eventually in preclinical models to assess for immunogenicity, including functional immune responses, similar to studies that validated *Pf* epitopes [36,110–112]. Continued refinement of this model with emerging *in vitro* and *ex vivo* datasets, including those in IEDB [102], will further enhance epitope prioritization. Collectively, TEpiNom provides a data-driven framework that supports efficient preclinical vaccine development through systematic refinement of candidate T-cell epitopes.

## Supplementary Material

MHC I HLA allele list

MHC II HLA allele list

Supplementary Tables S1-S8

## Figures and Tables

**Table 1:** MHC I and II alleles and allele groups associated with malaria-related outcomes. HLA alleles with positive or negative associations to distinct malaria outcomes as found through a PubMed literature search are shown here along with if they were associated with increased susceptibility to, increased protection against, or a mixed association with malaria outcomes. References for associated HLA alleles are shown in Supplementary Table 4.

	*MHC I Allele or Allele Group*	*MHC II Allele or Allele Group*

*Increased susceptibility to negative malaria outcomes*	A*01 A*20:01:01 A*29:02:01 A*30:01 A*33:01 A*66:02 B*53:01 C*06:02	DRB1*03 DRB1*13
	
*Increased protection against negative malaria outcomes*	B*35:01 B*53	DQB1*0501 DRB1*1302
	
*Mixed associations to different malaria outcomes (susceptibility and protection)*	-	DRB1*04 DRB1*10
	

## Data Availability

Data will be made available on request.

## Appendix A. Supplementary data

Supplementary data to this article can be found online at https://doi.org/10.1016/j.vaccine.2026.128388.
