# Supplementary material for: TEpiNom: A computational framework integrating population data to prioritize Plasmodium falciparum T cell epitopes
Source: Vaccine. Author manuscript; Available in PMC 2026 Apr 21. (PMC13097472; doi:10.1016/j.vaccine.2026.128388)
Supplement: Supplementary Tables S1-S8 [file NIHMS2162227-supplement-Supplementary_Tables_S1-S8.docx]

| Malaria Stage & Substage | Protein Name | Function | Reference |
| --- | --- | --- | --- |
| Skin to hepatocyte (Pre-erythrocytic) | CelTOS | Cell traversal | [40] |
|  | SPECT1 | Cell traversal | [41] |
|  | TLP | Motility and cell traversal | [42,43] |
|  | PL | Cell traversal | [44] |
|  | TRAP | Motility and sporozoite invasion | [45,46] |
|  | CSP | Motility, attachment, and regulation of host cell gene expression | [47,48] |
| Invasion of hepatocyte (Pre-erythrocytic) | RON4 | Tight junction formation during hepatocyte invasion | [49] |
|  | p36 | Commitment to hepatocyte infection | [50–52] |
|  | P36p/p52 | Commitment to hepatocyte infection | [50–52] |
|  | AMA1 | Invasion of hepatocyte | [53,54] |
|  | p24_1 | Trafficking of sporozoite invasion proteins | [55] |
|  | p24_2 | Trafficking of sporozoite invasion proteins | [55] |
|  | p24_3 | Trafficking of sporozoite invasion proteins | [55] |
|  | HSP70-2 | Molecular chaperone during hepatocyte invasion | [56] |
|  | TRSP | Invasion of hepatocyte | [57] |
| Development in hepatocyte (Pre-erythrocytic) | LSA1 | Liver stage differentiation | [58,59] |
|  | FabB/F | Liver stage development | [58,59] |
|  | FabZ | Liver stage development | [58,59] |
|  | FabG | Liver stage development | [58,59] |
|  | SLARP/SAP1 | Parasitophorous vacuole remodeling and Liver stage development | [62,63] |
|  | LISP1 | Merozoite release from liver stage | [64] |
|  | PDHEIa, PDHE3 | Fatty acid biosynthesis | [65] |
|  | PKG | Liver stage maturation | [66] |
|  | PALM | Segregation of merozoite during liver stage maturation | [67] |
|  | UIS3 | Liver stage development | [68] |
|  | UIS4 | Liver stage development | [69] |
|  | MIF | Liver stage development | [70] |
|  | ROM1 | Parasitophorous vacuole formation | [71] |
| Merozoite (Erythrocytic) | Ripr | Attachment and invasion of the red blood cell | [72,73] |
|  | MSP1 | Invasion of red blood cell | [74] |
|  | MSP3 | Invasion of red blood cell | [75] |
|  | GLURP | Attachment and invasion of the red blood cell | [76] |
|  | EBA-175 | Invasion of red blood cell | [77] |
|  | PfRh5 | Invasion of red blood cell | [73] |
|  | RON2 | Rhoptry and microneme formation and invasion of red blood cell | [78] |
| Other (Erythrocytic) | PfSEA1 | Egress from red blood cell | [79] |
|  | PfGARP | Cytoadherence of infected red blood cell | [80] |
| Placental (Erythrocytic) | VAR2CSA | Placental sequestration of merozoite | [81] |
| Sexual | Pfs25 | Ookinete survival | [82] |
|  | Pfs230 | Gametocyte fertilization and oocyst development | [83] |
|  | Pfs48/45 | Gametocyte fertilization and oocyst development | [83] |
|  | Pfs47 | Ookinete survival and immune evasion | [84] |

**Supplementary Table 1. Protein candidates and associated function.** Protein candidates included in this study divided by substage with described function(s) and primary reference demonstrating association. Summary of functional associations of liver protein candidates adapted from Duffy *et al.* [38].

| Malaria Stage & Substage | Protein Name | Number of Sequences Post-QC |
| --- | --- | --- |
| Skin to hepatocyte (Pre-erythrocytic) | CelTOS | 4049 |
|  | SPECT1 | 4045 |
|  | TLP | 1989 |
|  | PL | 3386 |
|  | TRAP | 3976 |
|  | CSP | 4035 |
| Invasion of hepatocyte (Pre-erythrocytic) | RON4 | 3996 |
|  | p36 | 3904 |
|  | P36p/p52 | 4004 |
|  | AMA1 | 4032 |
|  | p24_1 | 3955 |
|  | p24_2 | 4048 |
|  | p24_3 | 4018 |
|  | HSP70-2 | 4028 |
|  | TRSP | 4054 |
| Development in hepatocyte (Pre-erythrocytic) | LSA1 | 3930 |
|  | FabB/F | 4033 |
|  | FabZ | 4044 |
|  | FabG | 4008 |
|  | SLARP/SAP1 | 1760 |
|  | LISP1 | 2678 |
|  | PDHEIa PDHE3 | 3996 |
|  | PKG | 3962 |
|  | PALM | 4027 |
|  | UIS3 | 4054 |
|  | UIS4 | 4054 |
|  | MIF | 4051 |
|  | ROM1 | 4052 |
| Merozoite (Erythrocytic) | Ripr | 3884 |
|  | MSP1 | 1001 |
|  | MSP3 | 3953 |
|  | GLURP | 3978 |
|  | EBA-175 | 3744 |
|  | PfRh5 | 3659 |
|  | RON2 | 3797 |
| Other (Erythrocytic) | PfSEA1 | 2556 |
|  | PfGARP | 2808 |
| Placental (Erythrocytic) | VAR2CSA | 368 |
| Sexual | Pfs25 | 4054 |
|  | Pfs230 | 3413 |
|  | Pfs48/45 | 4037 |
|  | Pfs47 | 4044 |

**Supplementary Table 2. Protein sequence data set per malaria antigen.** Number of sample isolates in each protein’s sequence data set after quality control filtering. Sequences were acquired from MalariaGEN *Plasmodium falciparum* genomic data set and filtered out if within-infection fixation index (F_WS_) was greater than 0.95 indicating a potential polyclonal infection or if the resulting consensus sequence contained biologically improbable nonsense mutations.

| Malaria Stage & Substage | Protein Name | MHC I | | | MHC II | | |
| --- | --- | --- | --- | --- | --- | --- | --- |
|  |  | Pre-Filtration Epitope Count | Post-Filtration Epitope Count | Epitope Retention Rate (%) | Pre-Filtration Epitope Count | Post-Filtration Epitope Count | Epitope Retention Rate (%) |
| Skin to hepatocyte (Pre-erythrocytic) | CelTOS | 7403 | 36 | 0.49% | 3395 | 10 | 0.29% |
|  | SPECT1 | 4668 | 16 | 0.34% | 1669 | 5 | 0.30% |
|  | TLP | 17956 | 126 | 0.70% | 6361 | 42 | 0.66% |
|  | PL | 6934 | 84 | 1.21% | 2487 | 62 | 2.49% |
|  | TRAP | 5405 | 22 | 0.41% | 5915 | 3 | 0.05% |
|  | CSP | 7096 | 29 | 0.41% | 2972 | 5 | 0.17% |
| Invasion of hepatocyte (Pre-erythrocytic) | RON4 | 17725 | 72 | 0.41% | 6513 | 56 | 0.86% |
|  | p36 | 4007 | 95 | 2.37% | 1348 | 44 | 3.26% |
|  | P36p/p52 | 7117 | 70 | 0.98% | 2521 | 34 | 1.35% |
|  | AMA1 | 12969 | 57 | 0.44% | 6577 | 17 | 0.26% |
|  | p24_1 | 4413 | 51 | 1.16% | 1516 | 12 | 0.79% |
|  | p24_2 | 3026 | 39 | 1.29% | 1036 | 20 | 1.93% |
|  | p24_3 | 3041 | 42 | 1.38% | 1071 | 11 | 1.03% |
|  | HSP70-2 | 3947 | 64 | 1.62% | 1153 | 24 | 2.08% |
|  | TRSP | 2488 | 38 | 1.53% | 883 | 24 | 2.72% |
| Development in hepatocyte (Pre-erythrocytic) | LSA1 | 13957 | 19 | 0.14% | 5601 | 4 | 0.07% |
|  | FabB/F | 6303 | 53 | 0.84% | 2206 | 33 | 1.50% |
|  | FabZ | 4679 | 44 | 0.94% | 1679 | 9 | 0.54% |
|  | FabG | 4311 | 48 | 1.11% | 1495 | 20 | 1.34% |
|  | SLARP/SAP1 | 36450 | 270 | 0.74% | 12752 | 86 | 0.67% |
|  | LISP1 | 41658 | 584 | 1.40% | 14469 | 400 | 2.76% |
|  | PDHEIa PDHE3 | 9909 | 98 | 0.99% | 3478 | 58 | 1.67% |
|  | PKG | 5768 | 97 | 1.68% | 1797 | 61 | 3.39% |
|  | PALM | 3728 | 67 | 1.80% | 1259 | 68 | 5.40% |
|  | UIS3 | 2989 | 34 | 1.14% | 1000 | 20 | 2.00% |
|  | UIS4 | 2365 | 15 | 0.63% | 908 | 5 | 0.55% |
|  | MIF | 1063 | 13 | 1.22% | 335 | 3 | 0.90% |
|  | ROM1 | 2661 | 82 | 3.08% | 868 | 51 | 5.88% |
| Merozoite (Erythrocytic) | Ripr | - | - | - | 3649 | 45 | 1.23% |
|  | MSP1 | - | - | - | 5579 | 45 | 0.81% |
|  | MSP3 | - | - | - | 1573 | 5 | 0.32% |
|  | GLURP | - | - | - | 6269 | 11 | 0.18% |
|  | EBA-175 | - | - | - | 5966 | 65 | 1.09% |
|  | PfRh5 | - | - | - | 1918 | 38 | 1.98% |
|  | RON2 | - | - | - | 9417 | 241 | 2.56% |
| Other (Erythrocytic) | PfSEA1 | - | - | - | 8472 | 42 | 0.50% |
|  | PfGARP | - | - | - | 2129 | 0 | 0% |
| Placental (Erythrocytic) | VAR2CSA | - | - | - | 11360 | 52 | 0.46% |
| Sexual | Pfs25 | - | - | - | 476 | 15 | 3.15% |
|  | Pfs230 | - | - | - | 10517 | 194 | 1.84% |
|  | Pfs48/45 | - | - | - | 1240 | 34 | 2.74% |
|  | Pfs47 | - | - | - | 2205 | 18 | 0.82% |

**Supplementary Table 3. Predicted and retained T cell epitopes across malaria vaccine candidate proteins.** Number of predicted MHC I and MHC II epitopes before and after applying conservation and binding affinity filters (<10% median binding rank, >95% conservation). Data is categorized by malaria life cycle stage and substage with epitope counts shown for each protein after filtration steps. The percentage of retained epitopes after filtration is also provided for both MHC I and MHC II predictions as an epitope retention rate. MHC I epitope predictions performed exclusively for pre-erythrocytic stage proteins.

| a. MHC I Pre-erythrocytic Stage Epitope Combination Population Coverage | | | | | | | | | | |  |
| --- | --- | --- | --- | --- | --- | --- | --- | --- | --- | --- | --- |
| Epitope Combination | Epitope | Protein Name | Epitope Sequence Conservation | Population Coverage (%) | | | | | | | Coverage of High-Risk HLA Alleles |
|  |  |  |  | HLA-A Locus | HLA-B Locus | | HLA-C Locus | | Inter-Locus (MHC I) | |  |
| 1 | MAYNIWEEY | p36 | 0.997 | 74.95 | 79.65 | | 82.06 | | 99.09 | | A*29:02, A*66:02 |
| 2 | YLSPYPPVI | LISP1 | 0.997 | 87.99 | 84.37 | | 99.13 | | 99.98 | | A*29:02, A*66:02 |
|  | MAYNIWEEY | p36 | 0.997 |  |  |  |  |  |  |  |  |
| 3 | RTNLNSLFY | FabG | 0.999 | 96.2009 | 89.3938 | | 97.0981 | | 99.99 | | A*29:02, A*30:01, A*66:02 |
|  | YIMANFHNV | p36 | 0.997 |  |  |  |  |  |  |  |  |
|  | YEFICGPLPF | PKG | 1.000 |  |  |  |  |  |  |  |  |
| 4 | LPHRYPFLL | FabZ | 0.999 | 96.2009 | 98.4268 | | 97.0981 | | 100.0 | | A*29:02, A*30:01, A*66:02 |
|  | RTNLNSLFY | FabG | 0.999 |  |  |  |  |  |  |  |  |
|  | YIMANFHNV | p36 | 0.997 |  |  |  |  |  |  |  |  |
|  | YEFICGPLPF | PKG | 1.000 |  |  |  |  |  |  |  |  |
|  | | | | | | | | | | |  |
| b. MHC II Pre-erythrocytic Stage Epitope Combination Population Coverage | | | | | | | | | | |  |
| Epitope Combination | Epitope | Protein Name | Epitope Sequence Conservation | Population Coverage (%) | | | | | | | Coverage of High-Risk HLA Allele Groups |
|  |  |  |  | DRB1 Locus | DPA1-DPB1 Loci | | DQA1-DQB1 Loci | | Inter-Locus (MHC II) | |  |
| 1 | RKDFISFRITKLIKL | PL | 1.000 | 95.40 | 90.78 | | 48.06 | | 99.78 | | DRB1*04 (9/9) |
| 2 | RKDFISFRITKLIKL | PALM | 0.998 | 99.32 | 93.54 | | 92.87 | | 100 | | DRB1*04 (9/9) |
|  | MKIIIASSAAVAVLA | PL | 1.000 |  |  |  |  |  |  |  |  |
|  | | | | | | | | | | |  |
| c. MHC II Erythrocytic Stage Epitope Combination Population Coverage | | | | | | | | | | |  |
| Epitope Combination | Epitope | Protein Name | Epitope Sequence Conservation | Population Coverage (%) | | | | | | | Coverage of High-Risk HLA Allele Groups |
|  |  |  |  | DRB1 Locus | DPA1-DPB1 Loci | | DQA1-DQB1 Loci | | Inter-Locus (MHC II) | |  |
| 1 | QNKYVPINAVRVSRI | EBA-175 | 0.999 | 93.78 | 39.56 | | 77.89 | | 99.17 | | DRB1*04 (8/9) |
| 2 | QNKYVPINAVRVSRI | EBA-175 | 0.999 | 99.35 | 92.33 | | 86.35 | | 99.99 | | DRB1*04 (9/9) |
|  | VWKVISSFALHHLKN | RON2 | 0.999 |  |  |  |  |  |  |  |  |
| 3 | QNKYVPINAVRVSRI | EBA-175 | 0.999 | 99.49 | 97.12 | | 93.64 | | 100 | | DRB1*04 (9/9) |
|  | VWKVISSFALHHLKN | RON2 | 0.999 |  |  |  |  |  |  |  |  |
|  | KKRKYFLDVLESDLM | MSP1 | 1.000 |  |  |  |  |  |  |  |  |
|  | | | | | | | | | | |  |
| d. MHC II Sexual Stage Epitope Combination Population Coverage | | | | | | | | | | |  |
| Epitope Combination | Epitope | Protein Name | Epitope Sequence Conservation | Population Coverage (%) | | | | | | | |
|  |  |  |  | DRB1 Locus | | DPA1-DPB1 Loci | | DQA1-DQB1 Loci | | Inter-Locus (MHC II) | |
| 1 | HSYFIYDKIRLIIPK | Pfs48/45 | 1.000 | 81.39 | | 87.43 | | 15.06 | | 98.01 | |
| 2 | KTHFENFFVNPFNLK | Pfs230 | 1.000 | 95.66 | | 83.88 | | 75.02 | | 99.83 | |
|  | MMLYISAKKAQVAFI | Pfs48/45 | 0.999 |  |  |  |  |  |  |  |  |
| 3 | NGILYLKNNLANFTY | Pfs230 | 0.999 | 99.15 | | 87.83 | | 69.77 | | 99.97 | |
|  | KYAINSSFSDFYLKI | Pfs47 | 0.995 |  |  |  |  |  |  |  |  |
|  | HSYFIYDKIRLIIPK | Pfs48/45 | 1.000 |  |  |  |  |  |  |  |  |
| 4 | FLFIQLSIKYNNAKV | Pfs25 | 1.000 | 97.68 | | 87.89 | | 85.44 | | 99.96 | |
|  | IRSVLQSGALPSVGV | Pfs230 | 0.997 |  |  |  |  |  |  |  |  |
|  | KYAINSSFSDFYLKI | Pfs47 | 0.995 |  |  |  |  |  |  |  |  |
|  | HSYFIYDKIRLIIPK | Pfs48/45 | 1.000 |  |  |  |  |  |  |  |  |

**Supplementary Table 4. Epitope combinations for optimized MHC population coverage.** MHC I or MHC II epitope combinations within each malaria life cycle stage that maximize coverage of HLA alleles within the sub-Saharan African population are shown, after filtering by median binding affinity rank and epitope sequence conservation. The tables display the epitope combinations with the highest coverage as the number of epitopes in the combination increases until coverage reaches 100% or stagnates. Intra-locus coverage indicates the percentage of the population with a single HLA locus covered by the epitope combination, based on the HLA allele’s computed phenotypic frequencies, while inter-locus coverage indicates the percentage of the population covered across all MHC I or II loci by the epitope combination. The coverage by each epitope combination of high-risk HLA alleles associated with severe malaria outcomes was also shown. For MHC I, these high-risk alleles were HLA-A*29:02, HLA-A*30:01, HLA-A*33:01, and HLA-A*66:02. For MHC II, this was the allele group HLA-DRB1*04.

| a. MHC I Pre-erythrocytic Stage Epitope-dense Region Population Coverage | | | | | | | | | | | |  |
| --- | --- | --- | --- | --- | --- | --- | --- | --- | --- | --- | --- | --- |
| Genomic Window Size (aa) | Protein Name | | Region Coordinates (aa) | Number of Filtered Epitopes | Average Epitope Sequence Conservation | Population Coverage (%) | | | | | | Coverage of High-Risk HLA Alleles |
|  |  |  |  |  |  | HLA-A Locus | HLA-B Locus | | HLA-C Locus | Inter-Locus (MHC I) | |  |
| 50 | TRSP | | 2-51 | 8 | 0.999 | 98.73 | 91.94 | | 96.04 | 100 | | A*29:02, A*30:01, A*66:02 |
|  | p36 | | 15-64 | 8 | 0.999 | 97.13 | 97.38 | | 95.42 | 100 | | A*29:02, A*30:01, A*66:02 |
|  | ROM1 | | 1-50 | 6 | 0.999 | 93.83 | 96.40 | | 97.94 | 100 | | A*29:02, A*30:01, A*66:02 |
|  | LISP1 | | 3031-3080 | 8 | 0.999 | 96.63 | 94.49 | | 98.07 | 100 | | A*29:02, A*30:01, A*66:02 |
| 100 | p24_1 | | 210-309 | 4 | 0.999 | 94.68 | 74.59 | | 99.65 | 100 | | A*29:02, A*30:01, A*66:02 |
|  | RON4 | | 826-925 | 10 | 0.999 | 95.023 | 97.66 | | 95.84 | 100 | | A*29:02, A*30:01, A*66:02 |
|  | LISP1 | | 1175-1274 | 8 | 0.994 | 96.37 | 91.39 | | 98.72 | 100 | | A*29:02, A*30:01, A*66:02 |
|  | LISP1 | | 3117-3216 | 12 | 0.999 | 97.42 | 95.49 | | 96.99 | 100 | | A*29:02, A*33:01, A*66:02 |
| 150 | P36p/p52 | | 314-463 | 8 | 0.999 | 97.80 | 86.49 | | 98.56 | 100 | | A*29:02, A*30:01, A*66:02 |
|  | FabG | | 150-299 | 9 | 0.999 | 97.23 | 66.60 | | 99.32 | 100 | | A*29:02, A*30:01, A*66:02 |
|  | AMA1 | | 231-380 | 10 | 0.998 | 94.93 | 95.58 | | 98.50 | 100 | | A*29:02, A*30:01, A*66:02 |
|  | FabZ | | 42-191 | 8 | 0.999 | 99.05 | 97.71 | | 89.51 | 100 | | A*29:02, A*30:01, A*66:02 |
|  | LISP1 | | 773-922 | 8 | 0.999 | 97.84 | 97.57 | | 91.77 | 100 | | A*29:02, A*33:01, A*66:02 |
|  | PKG | | 458-607 | 10 | 0.999 | 97.62 | 90.72 | | 98.68 | 100 | | A*29:02, A*30:01, A*66:02 |
| 200 | LISP1 | | 3182-3381 | 7 | 0.999 | 96.80 | 97.17 | | 97.92 | 100 | | A*29:02, A*30:01, A*33:01  A*66:02 |
|  | |  | | | | | | | | | |  |
| b. MHC II Pre-erythrocytic Stage Epitope-dense Region Population Coverage | | | | | | | | | | | |  |
| Epitope Region | Protein Name | | Region (aa) | Number of Filtered Epitopes | Average Epitope Sequence Conservation | Population Coverage (%) | | | | | | Coverage of High-Risk HLA Allele Groups |
|  |  |  |  |  |  | DRB1 Locus | DPA1-DPB1 Loci | | DQA1-DQB1 Loci | Inter-Locus (MHC II) | |  |
| 50 | PALM | | 223-272 | 3 | 0.998 | 99.47 | 97.89 | | 82.24 | 100 | | DRB1*04 (9/9) |
|  | TLP | | 198-247 | 4 | 0.999 | 98.15 | 99.43 | | 76.57 | 100 | | DRB1*04 (9/9) |
|  | PL | | 10-59 | 5 | 0.999 | 99.53 | 96.44 | | 76.66 | 100 | | DRB1*04 (9/9) |
|  | SLARP/SAP1 | | 2834-2883 | 7 | 0.999 | 99.94 | 93.06 | | 67.44 | 100 | | DRB1*04 (9/9) |
|  | LISP1 | | 1459-1508 | 3 | 0.996 | 99.90 | 85.87 | | 97.74 | 100 | | DRB1*04 (9/9) |
| 100 | PL | | 570-669 | 3 | 1.000 | 99.96 | 99.24 | | 65.29 | 100 | | DRB1*04 (9/9) |
|  | SLARP/SAP1 | | 2576-2675 | 4 | 0.999 | 99.92 | 94.06 | | 44.23 | 100 | | DRB1*04 (9/9) |
|  | LISP1 | | 1945-2044 | 4 | 0.999 | 99.81 | 97.80 | | 68.19 | 100 | | DRB1*04 (9/9) |
| 150 | p24_2 | | 4-153 | 4 | 0.998 | 99.69 | 87.73 | | 88.35 | 100 | | DRB1*04 (9/9) |
|  | LISP1 | | 2230-2379 | 4 | 0.998 | 99.97 | 95.04 | | 53.49 | 100 | | DRB1*04 (9/9) |
|  | PKG | | 494-643 | 5 | 0.999 | 99.57 | 97.22 | | 77.60 | 100 | | DRB1*04 (9/9) |
| 200 | LISP1 | | 2831-3030 | 7 | 0.998 | 99.27 | 98.37 | | 61.501 | 100 | | DRB1*04 (9/9) |
|  | PKG | | 255-454 | 8 | 0.999 | 99.99 | 75.38 | | 65.91 | 100 | | DRB1*04 (9/9) |
|  | |  | | | | | | | | | |  |
| c. MHC II Erythrocytic Stage Epitope-dense Region Population Coverage | | | | | | | | | | | |  |
| Epitope Region | Protein Name | | Region (aa) | Number of Filtered Epitopes | Average Epitope Sequence Conservation | Population Coverage (%) | | | | | | Coverage of High-Risk HLA Allele Groups |
|  |  |  |  |  |  | DRB1 Locus | DPA1-DPB1 Loci | | DQA1-DQB1 Loci | Inter-Locus (MHC II) | |  |
| 50 | PfRh5 | | 60-109 | 4 | 0.999 | 99.71 | 98.11 | | 36.48 | 100 | | DRB1*04 (9/9) |
|  | MSP1 | | 798-847 | 5 | 0.999 | 99.99 | 13.02 | | 80.17 | 100 | | DRB1*04 (9/9) |
|  | RON2 | | 1765-1814 | 4 | 0.999 | 99.78 | 99.61 | | 39.91 | 100 | | DRB1*04 (9/9) |
| 100 | RON2 | | 1449-1548 | 5 | 0.99 | 99.51 | 97.37 | | 77.98 | 100 | | DRB1*04 (9/9) |
|  | |  | | | | | | | | | |  |
| d. MHC II Sexual Stage Epitope-dense Region Population Coverage | | | | | | | | | | | |  |
| Epitope Region | Protein Name | | Region (aa) | Number of Filtered Epitopes | Average Epitope Sequence Conservation | Population Coverage (%) | | | | | | |
|  |  |  |  |  |  | DRB1 Locus | | DPA1-DPB1 Loci | | | DQA1-DQB1 Loci | Inter-Locus (MHC II) |
| 50 | Pfs230 | | 1468-1517 | 6 | 0.999 | 99.93 | | 82.65 | | | 79.04 | 100 |
|  | Pfs48/45 | | 74-123 | 4 | 0.999 | 99.88 | | 99.56 | | | 30.88 | 100 |
| 100 | Pfs230 | | 833-932 | 8 | 0.998 | 88.49 | | 99.84 | | | 77.19 | 100 |
| 150 | Pfs230 | | 2922-3071 | 5 | 0.981 | 98.73 | | 98.81 | | | 71.67 | 100 |
| 200 | Pfs230 | | 2393-2692 | 7 | 0.999 | 97.53 | | 99.79 | | | 76.96 | 100 |

**Supplementary Table 5. Epitope-dense regions for optimized MHC population coverage.** Epitope-dense regions within each malaria life cycle stage that maximize coverage of HLA alleles in the sub-Saharan African population are shown, after filtering epitopes by median binding affinity rank and sequence conservation. Region optimization was performed using genomic window sizes of 50, 100, 150, and 200aa. For brevity, only the top performing regions that achieved 100% inter-locus population coverage and covered all high-risk HLA alleles are shown, or the regions that covered the largest possible subset of these high-risk alleles when complete coverage was not attained. Larger regions were omitted when they entirely encompass a smaller region already reported. Intra-locus coverage represents the percentage of the population with at least one presenting allele within a given locus, based on computed phenotypic frequencies, while inter-locus coverage represents the percentage of the population covered across all MHC I or II loci. Coverage of high-risk HLA alleles is also shown. For MHC I, these alleles were HLA-A*29:02, HLA-A*30:01, HLA-A*33:01, and HLA-A*66:02. For MHC II, this was the HLA-DRB1*04 allele group.

| a. MHC I RTS,S CSP Population Coverage | | | | | | | |
| --- | --- | --- | --- | --- | --- | --- | --- |
| RTS,S CSP Epitopes with Highest Coverage | | Start Coordinate (aa) | Epitope Sequence Conservation | Population Coverage (%) | | | |
|  |  |  |  | HLA-A Locus | HLA-B Locus | HLA-C Locus | Inter-Locus (MHC I) |
| VDENANANSA | | 301 | 0.145 | 0 | 22.98 | 0 | 22.98 |
| DENANANSA | | 302 | 0.145 | 0 | 18.50 | 0 | 18.50 |
| EPSDKHIKEY | | 315 | 0.164 | 6.36 | 9.08 | 0 | 14.86 |
| PSDKHIKEY | | 316 | 0.164 | 12.51 | 0 | 0 | 12.51 |
| KEYLNKIQNSL | | 325 | 0.127 | 0 | 14.38 | 0 | 14.38 |
| YLNKIQNSL | | 327 | 0.130 | 46.31 | 37.61 | 80.74 | 93.55 |
| KIQNSLSTEW | | 330 | 0.229 | 6.95 | 16.73 | 0 | 22.51 |
| IQNSLSTEW | | 331 | 0.591 | 7.04 | 15.20 | 0 | 21.18 |
| STEWSPCSV | | 333 | 1.000 | 0 | 0 | 14.48 | 14.48 |
| TEWSPCSVT | | 334 | 1.000 | 0 | 27.17 | 0 | 27.17 |
| TEWSPCSV | | 334 | 1.000 | 0 | 8.29 | 0 | 8.29 |
| TEWSPCSVTC | | 334 | 1.000 | 0 | 5.45 | 0 | 5.45 |
| RIKPGSANK | | 341 | 0.869 | 39.43 | 0 | 0 | 39.43 |
| RIKPGSANKPK | | 341 | 0.869 | 21.81 | 0 | 0 | 21.81 |
| DELDYANDI | | 356 | 0.149 | 0 | 9.03 | 0 | 9.03 |
| YANDIEKKI | | 368 | 0.536 | 0 | 15.61 | 65.32 | 70.73 |
| CKMEKCSSV | | 374 | 1.000 | 0 | 24.64 | 0 | 24.64 |
| CKMEKCSSVF | | 374 | 1.000 | 0 | 23.18 | 0 | 23.18 |
| KMEKCSSVF | | 375 | 1.000 | 0.72 | 21.64 | 29.77 | 45.36 |
| MEKCSSVF | | 376 | 1.000 | 0 | 21.30 | 0 | 21.30 |
| **RTS,S CSP Region Overall MHC I Population Coverage:** | | | | **82.48** | **96.31** | **96.01** | **99.97** |
|  |  | | | | | | |
| b. MHC II RTS,S CSP Population Coverage | | | | | | | |
| RTS,S CSP Epitopes with Highest Coverage | | Start Coordinate (aa) | Epitope Sequence Conservation | Population Coverage (%) | | | |
|  |  |  |  | DRB1 Locus | DPA1-DPB1 Loci | DQA1-DQB1 Loci | Inter-Locus (MHC II) |
| KHIKEYLNKIQNSLS | | 336 | 0.127 | 11.99 | 0 | 0.61 | 12.53 |
| HIKEYLNKIQNSLST | | 337 | 0.127 | 26.06 | 0 | 0.61 | 26.52 |
| IKEYLNKIQNSLSTE | | 338 | 0.127 | 26.06 | 0 | 0.61 | 26.52 |
| KEYLNKIQNSLSTEW | | 339 | 0.127 | 29.91 | 0 | 0.61 | 30.34 |
| EYLNKIQNSLSTEWS | | 340 | 0.130 | 25.24 | 0 | 0.61 | 25.70 |
| YLNKIQNSLSTEWSP | | 341 | 0.130 | 11.00 | 0 | 0 | 11.00 |
| GIQVRIKPGSANKPK | | 363 | 0.869 | 0 | 0 | 3.32 | 3.32 |
| IQVRIKPGSANKPKD | | 364 | 0.857 | 0 | 0 | 3.32 | 3.32 |
| QVRIKPGSANKPKDE | | 365 | 0.289 | 0 | 0 | 3.32 | 3.32 |
| VRIKPGSANKPKDEL | | 366 | 0.289 | 0 | 0 | 3.32 | 3.32 |
| **RTS,S CSP Region Overall MHC II Population Coverage:** | | | | **77.56** | **0** | **15.286** | **80.99** |

**Supplementary Table 6. Population coverage of the CSP genomic region included in RTS,S/AS01.** The 189aa (199-387) CSP genomic segment included in the RTS,S vaccine was evaluated using TEpiNom’s genomic regio coverage model. MHC I and II intra-locus and inter-locus population coverages were calculated using the same methods and HLA allele frequency datasets for sub-Saharan Africa as implemented previously. Coverage metrics are shown for the complete region as well as for the epitopes that contributed heavily to the overall coverage. Intra-locus coverage represents the percentage of the population with at least one presenting allele within a given locus, based on computed phenotypic frequencies, while inter-locus coverage represents the percentage of the population covered across all MHC I or II loci.

| a. MHC I Alleles or Allele Groups Associated with a Clinical Outcome | | | |
| --- | --- | --- | --- |
| Allele or Allele Group | Clinical Association | Region | Reference |
| A*01 | Increased risk of parasitemia | Ghana | [112] |
| A*20:01:01 | Increased risk of severe malarial anemia | Nigeria | [113] |
| A*29:02:01 | Increased risk of cerebral malaria | Nigeria | [113] |
| A*30:01 | Increased risk of cerebral malaria | Mali | [114] |
| A*33:01 | Increased risk of cerebral malaria | Mali | [114] |
| A*66:02 | Increased risk of cerebral malaria | Nigeria | [113] |
| B*35:01 | Decreased risk of parasitemia | Ghana | [112] |
| B*53 | Decreased risk of severe malaria; Decreased risk of cerebral malaria | Burkina Faso | [115] |
| B*53:01 | Increased risk of parasitemia | Uganda | [116] |
| C*06:02 | Increased risk of parasitemia | Uganda | [116] |

| b. MHC II Alleles or Allele Groups Associated with a Clinical Outcome | | | |
| --- | --- | --- | --- |
| Allele or Allele Group | Clinical Association | Region | Reference |
| DQB1*0501 | Decreased risk of severe malaria in children; decreased risk of reinfection in children | Gabon | [116,117] |
| DRB1*03 | Increased risk of malaria | Senegal | [118] |
| DRB1*04 | Decreased risk of parasitemia | Tanzania | [119] |
| DRB1*04 | Increased risk of severe malaria | Gabon | [120] |
| DRB1*04 | Increased risk of severe malaria in children | Ghana | [121] |
| DRB1*10 | Decreased risk of parasitemia | Tanzania | [119] |
| DRB1*10 | Increased risk of malaria | Senegal | [118] |
| DRB1*13 | Increased risk of malaria | Senegal | [118] |
| DRB1*1302 | Decreased risk of severe malaria in children | Gambia | [115] |

**Supplementary Table 7. MHC I and II alleles and allele groups associated with clinical outcomes.** HLA alleles with positive or negative associations to distinct malaria clinical outcomes as found through a PubMed literature search are shown here along with specific outcome, country from which the data originated, and literature reference.

| a. MHC I Alleles or Allele Groups Associated with a Clinical Outcome | | | |
| --- | --- | --- | --- |
| Allele or Allele Group | Clinical Association | Region | Reference |
| A*01 | Increased risk of parasitemia | Ghana | [112] |
| A*20:01:01 | Increased risk of severe malarial anemia | Nigeria | [113] |
| A*29:02:01 | Increased risk of cerebral malaria | Nigeria | [113] |
| A*30:01 | Increased risk of cerebral malaria | Mali | [114] |
| A*33:01 | Increased risk of cerebral malaria | Mali | [114] |
| A*66:02 | Increased risk of cerebral malaria | Nigeria | [113] |
| B*35:01 | Decreased risk of parasitemia | Ghana | [112] |
| B*53 | Decreased risk of severe malaria; Decreased risk of cerebral malaria | Burkina Faso | [115] |
| B*53:01 | Increased risk of parasitemia | Uganda | [116] |
| C*06:02 | Increased risk of parasitemia | Uganda | [116] |

| b. MHC II Alleles or Allele Groups Associated with a Clinical Outcome | | | |
| --- | --- | --- | --- |
| Allele or Allele Group | Clinical Association | Region | Reference |
| DQB1*0501 | Decreased risk of severe malaria in children; decreased risk of reinfection in children | Gabon | [116,117] |
| DRB1*03 | Increased risk of malaria | Senegal | [118] |
| DRB1*04 | Decreased risk of parasitemia | Tanzania | [119] |
| DRB1*04 | Increased risk of severe malaria | Gabon | [120] |
| DRB1*04 | Increased risk of severe malaria in children | Ghana | [121] |
| DRB1*10 | Decreased risk of parasitemia | Tanzania | [119] |
| DRB1*10 | Increased risk of malaria | Senegal | [118] |
| DRB1*13 | Increased risk of malaria | Senegal | [118] |
| DRB1*1302 | Decreased risk of severe malaria in children | Gambia | [115] |

**Supplementary Table 7. MHC I and II alleles and allele groups associated with clinical outcomes.** HLA alleles with positive or negative associations to distinct malaria clinical outcomes as found through a PubMed literature search are shown here along with specific outcome, country from which the data originated, and literature reference.

| a. Experimentally validated and Down-selected MHC I Epitopes | | | | |
| --- | --- | --- | --- | --- |
| Malaria Stage | Protein Name | Number of Experimentally Validated, Down-selected Epitopes | T Cell Result | Reference |
| Pre-erythrocytic | CelTOS | 13 | IFNγ release | [109,122] |
|  | TRAP | 41 | IFNγ release, cytotoxicity, IL-10 release, proliferation, IL-5 release, granzyme B release, | [111,123–137] |
|  | CSP | 40 | Proliferation, cytotoxicity, TNFa release, IFNγ release, IL-4 release, IL-2 release, IL-10 release, IL-5 release, antibody help | [111,123,124,126,128,128,131,132,136,138–163] |
|  | AMA1 | 38 | Proliferation, granzyme B release, IFNγ release, | [48,111,126,127,148,151,164–170] |
|  | LSA1 | 8 | IFNγ release, proliferation, qualitative binding | [136,171–175] |
|  | UIS3 | 5 | IFNγ release | [174] |
| b. Experimentally validated and Down-selected MHC II Epitopes | | | | |
| Malaria Stage | Protein Name | Number of Experimentally Validated, Down-selected Epitopes | T Cell Result | Reference |
| Pre-erythrocytic | CelTOS | 2 | IFNγ release | [109] |
|  | TRAP | 2 | IFNγ release, proliferation, IL-5 release, IL-10 release, IL-4 release | [123,128,130,134–137] |
|  | CSP | 3 | IFNγ release, proliferation, IL-10 release | [123,128,136,141–144,146,151,152,154,158] |
|  | AMA1 | 1 | IFNγ release | [127,170] |
|  | UIS3 | 2 | IFNγ release | [174] |
| Erythrocytic | MSP1 | 4 | IFNγ release, proliferation, IL-10 release, IL-13 release | [176–178] |
|  | MSP3 | 2 | IFNγ release, proliferation, IL-10 release, IL-6 release, TNF release | [179–184] |
|  | EBA-175 | 1 | IFNγ release, proliferation, IL-4 release | [185] |
|  | PfRh5 | 4 | IFNγ release | [48] |

**Supplementary Table 8. Protein candidates and published experimental validation of T cell epitopes.** Protein candidates included in this study divided by stage with described studies showing T cell epitope validation.
